# Transcriptomic analysis of grape (*Vitis vinifera* L.) leaves during and after recovery from heat stress

**DOI:** 10.1186/1471-2229-12-174

**Published:** 2012-09-28

**Authors:** Guo-Tian Liu, Jun-Fang Wang, Grant Cramer, Zhan-Wu Dai, Wei Duan, Hong-Guo Xu, Ben-Hong Wu, Pei-Ge Fan, Li-Jun Wang, Shao-Hua Li

**Affiliations:** 1 Institute of Botany, Chinese Academy of Sciences, Beijing, 100093, P.R. of China; 2 University of Chinese Academy of Sciences, Beijing, 100049, P.R. of China; 3 Department of Biochemistry and Molecular Biology, University of Nevada, Reno, 89557, USA; 4 INRA, ISVV, UMR 1287 EGFV, Villenave d'Ornon, 33882, France; 5 Key Laboratory of Plant Germplasm Enhancement and Speciality Agriculture, Wuhan Botanical Garden, Chinese Academy of Sciences, Wuhan, 430074, P.R. of China

## Abstract

**Background:**

Grapes are a major fruit crop around the world. Heat stress can significantly reduce grape yield and quality. Changes at the molecular level in response to heat stress and subsequent recovery are poorly understood. To elucidate the effect of heat stress and subsequent recovery on expression of genes by grape leaves representing the classic heat stress response and thermotolerance mechanisms, transcript abundance of grape (*Vitis vinifera* L.) leaves was quantified using the Affymetrix Grape Genome oligonucleotide microarray (15,700 transcripts), followed by quantitative Real-Time PCR validation for some transcript profiles.

**Results:**

We found that about 8% of the total probe sets were responsive to heat stress and/or to subsequent recovery in grape leaves. The heat stress and recovery responses were characterized by different transcriptional changes. The number of heat stress-regulated genes was almost twice the number of recovery-regulated genes. The responsive genes identified in this study belong to a large number of important traits and biological pathways, including cell rescue (i.e., antioxidant enzymes), protein fate (i.e., HSPs), primary and secondary metabolism, transcription factors, signal transduction, and development. We have identified some common genes and heat shock factors (HSFs) that were modulated differentially by heat stress and recovery. Most HSP genes were upregulated by heat stress but were downregulated by the recovery. On the other hand, some specific HSP genes or HSFs were uniquely responsive to heat stress or recovery.

**Conclusion:**

The effect of heat stress and recovery on grape appears to be associated with multiple processes and mechanisms including stress-related genes, transcription factors, and metabolism. Heat stress and recovery elicited common up- or downregulated genes as well as unique sets of responsive genes. Moreover, some genes were regulated in opposite directions by heat stress and recovery. The results indicated HSPs, especially small HSPs, antioxidant enzymes (i.e., ascorbate peroxidase), and galactinol synthase may be important to thermotolerance of grape. HSF30 may be a key regulator for heat stress and recovery, while HSF7 and HSF1 may only be specific to recovery. The identification of heat stress or recovery responsive genes in this study provides novel insights into the molecular basis for heat tolerance in grape leaves.

## Background

Most crop plants are exposed to heat stress during certain stages of their life cycle. Heat stress, defined as the temperature above a normal optimum, is expected to become a major issue in reducing crop production in coming years due to global warming [1]. Grape is a popular cultivated fruit throughout the world and represents one of the most important crops with highly valued products such as juices, liquors and wines [2]. The grape species *Vitis vinifera* makes up most of the grape production in the world. However, grape production and quality often fluctuate due to various environmental factors. Temperature has been broadly considered as a major determining factor. Studies show that crop production is severely limited by temperature stresses around the world [3]. In many regions, the maximum midday air temperature can reach 40°C and above, which can destroy grape berry ripening [4]. In addition, crop cultivation in sheltered conditions (e.g., greenhouses and hoop houses) is common in many regions. These conditions can further increase temperature due to inadequate air circulation. Studies indicated that temperatures above 35°C generally reduce photosynthesis in grape leaves [5]. Extreme temperatures may endanger berry quality and economic returns [4,6]. This is expected to get worse with more frequent high temperature stress due to climate change [3,7].

In the past, studies of response and adaptation of grape to high temperatures have focused mostly on grape morphological and physiological changes including photosynthesis, respiration, cell membrane stability, hormone changes and antioxidant systems [6,8-13]. With the availability of the grape genome sequence [14,15], study of the functional genomics of grapes has become possible [3,16]. Transcriptomic analysis represents one of these major research opportunities. Gene expression is tissue- and development-specific. To date, transcriptomic studies in grapes has been primarily focused on berry development and water stress responses [17-29]. There are few transcriptomic reports of the effect of heat stress on grape.

Transcriptomic studies of heat stress effects on Arabidopsis, rice, tobacco, potato, tomato and sunflower have been reported [30-34]. Lim et al. [30] found that acclimating *Arabidopsis thaliana* suspension cells at a moderate heat enhanced heat resistance. Expression of 165 genes changed, especially those of heat shock proteins (HSPs). With cDNA microarrays and RT-qPCR techniques, Frank et al. [33] found that HSP70, HSP90, and heat shock transcription factors(HSF) HSFA2 and HSFA3 were important to tomato microspore resistance to heat stress. However, data on the molecular mechanisms involved in heat stress responses and thermotolerance in grape leaves are very limited.

Some studies have shown that the recovery process from heat stress in plants is very important to survival [35]. The degree of recovery from stress is a direct index of plant heat tolerance. Thus, there should be some differences between the recovery mechanisms and the direct heat response mechanisms in plants [36]. Gu et al. [37] found that sucrose synthase, calmodulin, certain peptides, and aquaporin genes in *Populus* were transcriptionally-activated only during recovery from salt stress.

In this study, we used oligonucleotide microarrays and quantitative Real-Time PCR (qRT-PCR) to identify genes in grape leaves with altered transcript accumulation during heat stress and after recovery to provide clues to the function of these genes during heat stress and subsequent recovery.

## Results

### Expression and validation of probes sets responsive to heat stress or recovery in grape leaves

**Figure 1:**
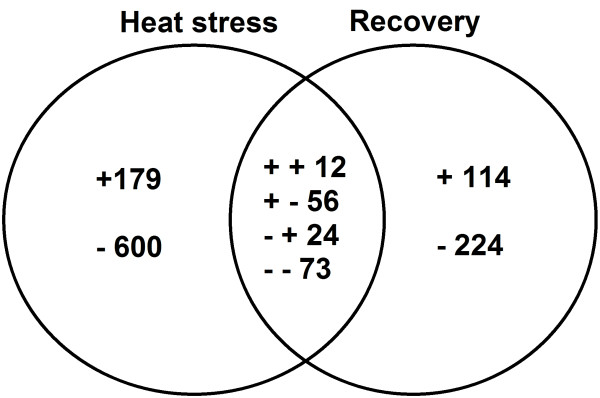
**Venn diagram of transcripts (both identified and unknown) that were up- or downregulated by heat stress or recovery.** The " + " and "-" indicate up- and downregulated transcripts, respectively. A total of 1282 transcripts were significantly (dChip, P < 0.001) affected by heat or recovery. 179: unique upregulated transcripts by heat stress; 114: unique upregulated transcripts by the recovery; 600: unique downregulated transcripts by heat stress; 224: unique downregulated transcripts by the recovery; 12: commonly upregulated transcripts by heat stress and recovery; 73: commonly downregulated transcripts by heat stress and recovery; 24: downregulated by heat stress but upregulated by recovery; 56: upregulated by heat stress but downregulated by recovery.

**Figure 2:**
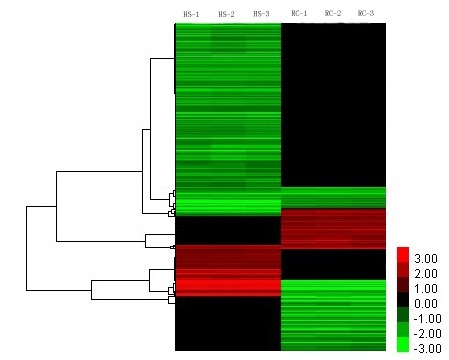
**Average linkage hierarchical clustering analysis of the Log**_**2**_**transformed values of fold changes of the 821 annotated genes during heat stress and after the following recovery.** HS-1, HS-2 and HS-3 represent three replications of heat stress treatments; RC-1, RC-2 and RC-3 represent three replications of the recovery.

Transcriptomic profiles of gene expression variation in *Vitis vinifera* cv. Cabernet Sauvignon leaves in response to heat treatment and subsequent recovery was quantitatively assessed using the Affymetrix Grape Genome Array with 15,700 probe sets. Array data were averaged for three biological replicates and filtered as described in Materials and Methods. Using the filtering criteria, 1282 (about 8% of total probe sets) were significantly affected by heat or recovery and were further analyzed. Heat stress and recovery affected transcript levels in different ways. Among those probe sets showing differential expression during heat stress (dChip, *P* < 0.001), 247 were upregulated, 697 were downregulated, while 150 were induced and 353 were repressed after recovery compared to their corresponding control levels (Figure 1). A total of 12 probe sets were commonly upregulated by heat stress and recovery, whereas 179 and 114 were specifically induced by both treatments, respectively. A total of 73 probe sets were commonly downregulated by both treatments, whereas 600 and 224 were specifically repressed by heat stress and recovery, respectively. Moreover, 24 probe sets were repressed by heat stress but induced by recovery, and 56 probe sets were upregulated by heat stress and downregulated by recovery. Cluster analysis also indicated that some genes were up- or downregulated under heat stress and after recovery (Figure 2). In order to validate the results obtained with the microarray analyses, we carried out qRT-PCR assays on 12 cDNA sequences using gene-specific primers (Additional file 1). The qRT-PCR profiles were analyzed on three biological replicates. Linear regression analyses displayed highly significant correlations (r = 0.982) between qRT-PCR and microarray results for the 12 evaluated genes (see Additional file 2), confirming the validity of the microarray results.

### Functional analysis of probes sets responsive to heat stress or recovery in grape leaves

**Figure 3:**
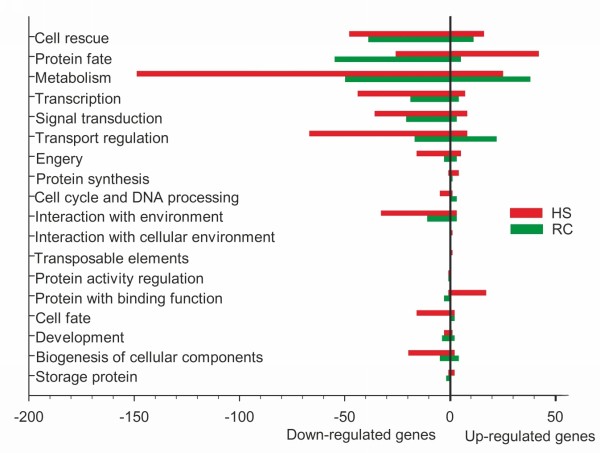
**Functional classification of heat stress and recovery–responsive transcripts.** Transcripts with a mean absolute expression ratio of at least 2.0 (linear scale) and a *P*<0.001 in a *t*-test for significance were classified into the categories shown, based on shared putative function and/or common structural motifs. HS heat stress; RC recovery.

There were a large number of genes which were responsive to heat stress or recovery that do not match any genes with known functions. Eight hundred and twenty-one probe sets were assigned to annotated genes and ESTs in PLEXdb ( http://www.plexdb.org). Functional categories of these probe sets based on MIPS (Munich Information Center for Protein sequences) are shown in Figure 3. Furthermore, we compared the common probe sets between heat stress and recovery, and analyzed probe sets unique to both treatments, based on functional classification. The changes of some genes such as HSPs were similar to those in previous studies on microarray analysis of heat stress in rice, Arabidopsis, tomato, barley and potato [30,31,33,38-41].

### Comparative analysis of common responsive probe sets between heat stress and subsequent recovery

There were 40 annotated genes that were upregulated by heat treatment, but were downregulated following recovery. They are listed in groups according to their putative involvement in different cellular events (see Additional file 3). Sixty percent of them were HSPs/molecular chaperones and may be critical to heat tolerance. The genes of high and middle molecular HSPs included *HSP62.6, HSC71.5, HSP70, HSP 101, HSP80.1*, low molecular HSPs genes included *CPN10*, *HSP11.1, HSP15.7, HSP16.1, HSP17.5, HSP17.6, HSP18.3, HSP18.6, HSP22, HSP25.7, HSP37.1* and *HSP40*. Interestingly, gene *HSF30* was upregulated 11-fold by heat stress, but downregulated more than 10-fold following recovery compared with the corresponding controls. In addition, genes related to cell rescue including *Hin1*, *Stil* and galactinol synthase changed. Three calmodulin-related proteins that suppress posttranscriptional gene silencing in plants were also involved in the process. Also affected by heat stress were genes of ethylene-responsive transcriptional co-activator-like protein, metabolism-associated enzymes, 3-hydroxy-3-methylglutaryl coenzyme A (acetyl-CoA pathway) and ER1(pyridoxin biosynthesis protein), development -related enzymes, and ripening regulated protein.

There were a total of 10 annotated genes upregulated by heat stress and subsequent recovery (see Additional file 4). *HSP20* showed a larger degree of up-regulation by heat stress than by recovery. The other genes were related to metabolism, transcription, cell rescue and transposable elements.

Additional file 5 shows 19 genes which were downregulated by heat stress but upregulated during subsequent recovery. Genes related to cell rescue included peroxidase 42 and alcohol dehydrogenase 7. A number of genes were related to metabolism; for example, genes involved in starch biosynthesis (ADP-glucose pyrophosphorylase large subunit 1), polysaccharide degradation (beta-galactosidase BG1), and polyphenol oxidase (tyrosine metabolism). Aquaporin and triose phosphate were affected and are related to transport regulation. In addition, the affected gene F14J22.4 is involved in signal transduction. No genes in the regulatory factor categories were identified.

Additional file 6 shows 53 genes that were downregulated by heat stress and subsequent recovery. Twelve of these genes were related to cell rescue including stress responses. Two Class IV chitinase transcripts were downregulated about 30-fold by heat treatment, but only downregulated 3-fold after the recovery. Other downregulated genes were involved in metabolism, signal transduction, transcript regulation and cell transport. Secondary metabolism genes that were downregulated included stilbene synthase, flavonol 3-O-glucosyltransferase 2, caffeic acid O-methyltransferase. Regulatory genes including WRKY transcription factor-b, Myb-related transcription factor MybB1-2, SPF1 protein (transcription activation), and NAC domain protein NAC1 were also downregulated.

### Analysis of unique probe sets responsive to heat stress in grape leaves

In Additional file 7, a total of 78 upregulated probe sets identified as unique to the heat stress response are listed. Stress-responsive genes affected included those for one salt-inducible protein, one wound-induced protein, a dehydroascorbate reductase, and a cytosolic ascorbate peroxidase (APX). There were nine HSPs/molecular chaperones influenced, including HSP21 which was upregulated 43-fold. Metabolism genes affected included tetrahydropicolinate succinylase, pectinesterase, homocysteine S-methyltransferase 2, putative ripening-related protein sterol 4-alpha-methyl-oxidase and adenine phosphoribosyl transferase. Four genes involved in signal transduction were affected including those for GTP-binding, a ADP-ribosylation factor-like protein, a receptor associated protein (transmembrane signal transduction) and strubbelig receptor family 3. Regulatory genes responding included genes for zinc finger protein-like, Myb-related protein, and triptychon. Metabolism genes affected included those for fatty acid biosynthesis.

There were 600 downregulated probe sets uniquely responsive to heat stress (Figure 1), of which 412 were identified (see Additional file 7). Stress response genes included those for chitinase, pathogenesis-related protein, osmotic, and cold stress response proteins. A total of 129 genes related to primary and secondary metabolism were downregulated by heat treatment. The transcript level of 13 genes involved in respiration and photosynthesis declined. Transcription regulatory genes that were down-regulated included those for zinc finger protein, pathogenesis-related gene transcriptional activator PTI5, Myb-related transcription factor VlMYBB1-2, ethylene response factor ERF3a, bHLH106, and MYC transcription factor. Down-regulated genes involved in transport regulation included cation, carbohydrate, amino acid, protein and nucleotide transporters.

### Analysis of unique responsive probe sets to recovery from heat stress

There were 72 upregulated probe sets unique to the recovery response (see Additional file 8). Stress genes that were upregulated included adenylyl-sulfate reductase precursor, Fe-SOD, late embryogenesis abundant protein D-29, and putative pathogenesis-related protein. Upregulated genes involved in protein fate only included U-Box protein and aspartyl protease family protein, but no HSPs were observed. There were 27 genes related to metabolism that were upregulated, involving carboxylate metabolism, biosynthesis of phenylalanine, phosphate metabolism, polysaccharide degradation, and fatty acid biosynthesis. There was only one signal transduction gene, a receptor protein kinase and a protein ralf-like 34 that were upregulated. There were 17 transporter genes upregulated after the recovery, including a nitrate transporter, a tonoplast intrinsic protein and several ABC transporters. Transcript regulatory genes upregulated including a squamosa promoter binding-like protein, a Myc-like anthocyanin regulatory protein, a putative Zinc finger protein, and a translation initiation factor IF2.

In addition, 137 downregulated probe sets were unique to the recovery response (see Additional file 8). Stress- responsive proteins that were downregulated were mainly defence proteins. Downregulated genes involved in protein fate included HSPs, HSFs (HSF1, HSF7), and protein kinase domain-containing protein. Downregulated genes related to metabolism numbered 34, and were involved in biosynthesis of glutamine, stilbenes, flavonoids and metabolism of proline and carbohydrates. Some downregulated genes were related to Ca^2+^ mediated signal transduction, and some were regulatory genes including NAC domain proteins, WRKY transcription factors, and Zinc finger DNA-binding protein.

## Discussion

High temperature disturbs cellular homeostasis in plants and can lead to severe retardation of growth and development and even death. Plants exposed to heat stress exhibit a characteristic set of cellular and metabolic responses. Based on the results detailed above, the discussion focuses on the response of grape leaves to heat stress and subsequent recovery from the following aspects.

### HSPs and antioxidant enzymes

One typical response to heat stress is an accelerated transcription of a set of stress or protein fate-related genes, such as those encoding HSPs, which are the main constituents of the heat shock response [1]. The major HSPs belong to five structurally distinct classes: Hsp100, Hsp90, Hsp70, Hsp60 and small HSPs (sHSPs) [42]. In this study, heat stress increased the expression of various HSPs. The transcript level of these HSPs decreased to the control level or even lower after the subsequent recovery (see Additional files 3 and 7). Under heat stress, many proteins in the cell are subject to denaturation, and HSPs function as molecular chaperones to provide protection. After the recovery, these HSPs are degraded. Moreover, a large amount of HSPs in cells resulted in the decline of transcript level of *HSPs* genes [43]. Wang et al. [44] reported that the expression of protein HSP21 was induced by heat stress in grapevine leaves, and was lower than that of the control after the recovery. In plants, sHSPs form a more diverse subfamily than other HSPs/chaperones with respect to sequence similarity, cellular location and function. The sHSPs are not themselves able to refold non-native proteins, but have a high capacity to bind non-native proteins, possibly through hydrophobic interactions, and to stabilize and prevent non-native aggregation, thereby facilitating their subsequent refolding by ATP-dependent chaperones such as the DnaK system or ClpB/DnaK. Increasing evidence suggests strong correlation between sHSP accumulation and plant tolerance to stress [45]. Chloroplast and mitochondrial sHSPs are considered to play an important role in heat tolerance [46,47]. In this study, HSP22 located in mitochondria was highly expressed under heat stress and was downregulated after recovery. HSP21 located in the chloroplast was highly expressed (43-fold) by heat stress, but its transcription level declined to the control level after the following recovery (see Additional file 7). This result shows that some sHSPs may have important effects on heat tolerance of grapevines.

Among the other HSPs, over expression of HSP101 in Arabidopsis had a positive effect on growth after recovery [36]. It was recently found that an HSP101 homologue in Arabidopsis was involved in conferring thermotolerance to chloroplasts during heat stress [48]. Genetic analysis in Arabidopsis indicated that HSP101 interacts with the sHSP chaperone system to re-solubilize protein aggregates after heat stress [49]. Recently, it was demonstrated that the transcript level of HSP101 increased in maturing tomato pollen grains in response to heat stress [33,50]. In the present study, HSP101 in grape leaves was upregulated by heat stress, exhibiting a 9-fold elevated expression, but it was downregulated 6-fold after the subsequent recovery. HSP70 has essential functions in preventing aggregation and in assisting refolding of non-native proteins under both normal and stress conditions [51]. Some family members of HSP70 are consistently expressed and are often referred to as HSC70. These members are often involved in assisting the folding of *de novo* synthesized polypeptides and the import/translocation of precursor proteins. In this study, the expression of HSC70 was induced by heat stress and declined after the subsequent recovery, in agreement with previous results.

In heat stress studies, increasing attention has being paid to the generation of reactive oxygen species (ROS) and the cellular antioxidant defense systems. ROS levels are controlled by a network of enzymes and metabolites, including superoxide dismutases (SOD), ascorbate peroxidase (APX), guaiacol peroxidase (GPX) and dehydroascorbate reductase (DHAR). APX plays a pivotal role in ROS metabolism. It catalyzes the reduction of hydrogen peroxide to water by using ascorbate as a specific electron donor [52]. APX appears to be regulated by HSFA2 [53]. Previous studies also indicated that it is involved in survival from high light stress [54]. The results of the present study showed that expression of *APX*, *peroxidase* 42 and *DHAR* were upregulated by 5-, 3.27- and 3.18-fold, respectively, indicating that these genes may have an important role in grape leaves in response to heat stress.

### Metabolism

Temperature is one of the most active environmental factors affecting all plant metabolic activities, including amino acid and carbohydrate metabolism [55]. Secondary metabolites are involved in resistance against heat shock [56]. In this study, galactinol synthase transcript level increased significantly (about 50-fold) in response to heat stress and declined during recovery. Heat shock induced the production of sugars including raffinose and galactinol [57]. The raffinose family of oligosaccharides has been implicated in the scavenging of hydroxyl radicals [58], and in protecting liposomes from desiccation through direct sugar–membrane interactions in soybean [59]. Galactinol synthase catalyzes the first step in the synthesis of raffinose polysaccharide, and is also regulated by HSFA2 in Arabidopsis, linking heat shock proteins and raffinose metabolism [60]. Weston et al. [61] reported that expression of AtGolS1 was observed even at optimal temperature, and was upregulated during heat stress. Several other probe sets representing genes related to carbohydrate metabolism were regulated by heat stress in our experiment, such as invertase, UDP-glucose dehydrogenase, 6-phosphate dehydrogenase, sucrose synthase, amylase and trehalose-6-phosphate synthase/phosphatase (see Additional files 3 and 7). These enzymes are important in sugar metabolism, which were downregulated by heat stress in this study. In addition, lipid metabolism was also inhibited by heat stress, with effects on lipase, L-asparaginase, lipoxygenase and fatty acid hydroperoxide lyase.

### Transcription factors (TFs)

HSFs play important roles in both basal and acquired thermotolerance through binding to *cis*-acting regulatory elements called heat shock element (HSEs) in the promoter region of HSP genes [62]. In general, plant HSFs are divided into three classes, HSFAs, HSFBs and HSFCs [63]. HSF30 belongs to HSFA2 which was the dominant HSF in thermotolerant cells [39,64] and was highly heat shock upregulated in mature tomato microspores [33]. The transcript levels of the HSFA2 target genes (e.g. *HSP101*, *HSP70*, *HSP22*, *HSP17.6*, and *HSP15.7*) were highly correlated with those of HSFA2 in *Pro35S:HSFA2* Arabidopsis plants, and the induction of HSFA2 target genes was strongly reduced under heat stress in HSFA2 knockout Arabidopsis plants [60]. In this study, *HSF30* exhibited increased expression levels (11-fold) under heat stress and was downregulated (14.3-fold) after the subsequent recovery corresponding to 16 HSPs (see Additional file 3). The present results indicated that HSF30 may also play an important regulatory role in the thermotolerance of grape. HSF7 is an HSFb2b and was strongly induced by heat stress in Arabidopsis, maize and tomato [64]. However, *HSF7* was weakly upregulated (1.7-fold) by heat stress, but showed significant down-regulation (3.7-fold) after recovery in the present study. This indicated that HSF7 may play an important role in reducing expression of HSPs after recovery in grape leaves. In addition, HSF1 governs the expression of HSPs and regulates thermotolerance [33,65]. HSF1 belongs to the HSFA1a group, which was identified as a master regulator of thermotolerance in tomato [64]. The synthesis of members of HSP100, HSP90, HSP70, HSP60 and sHSPs under heat stress in the HSF1-RNAi strains of Chlamydomonas was dramatically reduced or completely abolished [66]. In the present study, *HSF1* showed similar sign of change as *HSF7*. Therefore, HSF7 and HSF1 may play important roles in the recovery process from heat stress in grape leaves.

Qin et al. [67] reported that ethylene-responsive transcriptional co-activator (ERTCA) gene is upregulated more than 8-fold in all heat treated wheat leaves. Over-expression of *ERTCA* enhanced heat stress tolerance of Arabidopsis [68]. Heat stress induced expression of *ERTCA* rapidly in the sensitive genotype of tomato [38]. In the present study, the strong induction of *ERTCA* expression by heat stress in grape leaves provided another piece of evidence for its role in heat tolerance. Many other transcription factor genes were also affected by heat stress and recovery in grape leaves, although their roles in heat tolerance are not clear. Interestingly, the majority of heat response transcription factors genes were responsive to the heat treatment. Most of them were downregulated. Basic leucine zipper (bZIP) transcription factors play a role in plant pathogen responses, light signaling, and ABA and abiotic stress signaling [69]. Our microarray data revealed that bZIP transcription factors were heat-regulated. Some were upregulated, such as Zinc finger protein-like, some were downregulated, such as B-box type Zinc finger-containing protein. GATA-type Zinc finger protein was upregulated only by the recovery treatment (see Additional file 8). Plant transcription factors WRKYs have been reported in both biotic and abiotic stress responses [70]. In this study, three WRKY transcription factors were downregulated by heat stress, and two WRKY transcription factors were downregulated by the recovery (see Additional files 7 and 8). These results suggested that several WRKY factors could be involved in the heat response of grape leaves. Our data indicated that expression of 3 transcription factors with NAC domain genes were also heat–regulated. Plant-specific NAC family transcription factor has a conserved NAC domain at the N-terminal of the protein and has been implicated in plant development [71]. It was recently reported that several NAC transcription factors were also involved in biotic and abiotic stress response [72].

### Signal transduction components

In our study, 4 genes for receptor-like kinases (RLKs) were regulated by heat stress (see Additional file 7). RLK1 is induced by wounding, pathogen attack, and salicylic acid [73]. Recent work indicated that RLK1 plays an important role in abscisic acid (ABA) signal transduction [74]. Our results suggested that heat signal transduction in grape leaves shared, at least in part, some common pathways with other biotic, abiotic and ABA stress signaling through these RLKs. Protein phosphorylation and dephosphorylation have been reported in heat signal transduction. Indeed, protein kinases and phosphatases with altered expression formed the largest group of genes. In addition, many protein phosphatases showed differential expression, suggesting that protein post-translation modification occurred during the heat response of grapevine leaves.

Calcium is a universal signaling molecule in both animals and plants, and the transient increase of Ca^2+^ level during heat stress is well-documented in plants [11,75,76]. Heat shock triggered cytosolic Ca^2+^ bursts, which is transferred by Ca^2+^ binding proteins (CBP) such as calmodulin (CaM), CaM-related proteins, Ca^2+^-dependent protein kinases (CDPK), and calcineurin B-like protein (CBL), and then unregulated the expression of HSPs, due to the dependence of the final step in HSF- mediated HSP expression on a Ca^2+^ signal [77,78]. In the present analysis, candidate genes encoding the components of calcium- or calmodulin-mediated signal pathways, including calnexin, calmodulin, and CDPKs, were also heat- or recovery-regulated, suggesting a role of Ca^2+^-mediated signals in the heat stress response. Plant survival after severe environmental stress largely depends on the efficiency of recovery mechanisms. Among the genes activated after recovery, we found a calmodulin gene. qRT-PCR indicated a 4.28-fold induction of calmodulin transcript accumulation after the early response to salt stress [79].

## Conclusion

The data presented here provides genome-wide expression profiles of grape leaves under heat stress and subsequent recovery. Affymetrix Grape Genome Array and qRT-PCR techniques were used to identify heat stress- and recovery-regulated genes which represented classic heat stress responsive and thermotolerance mechanisms. The present study highlighted the significant contribution and fundamental roles of transcriptional control in stress responses in grape leaves. We found that about 8% of total probe sets were responsive to heat stress and subsequent recovery in grape leaves. Heat stress and recovery responses displayed differential gene expression changes. The number of heat stress-regulated genes was almost twice that of recovery-regulated genes. The responsive genes identified in this study belong to a large number of important factors and biological pathways, including those for cell rescue (i.e., antioxidant enzymes), protein fate (i.e., HSPs), primary and secondary metabolism, transcription factors, signal transduction and development. Of particular interest, HSPs especially sHSPs, APX and galactinol synthase, may be very important to thermotolerance of grape leaves; HSF30 may be a key regulator for heat stress and recovery;, and, HSF7 and HSF1 may mainly function after recovery. These results provide novel insight into the grape leaf response to heat stress and have great implications for further studies on gene function annotation and molecular breeding.

## Methods

### Plant materials and treatments

Stem cuttings of ‘Cabernet Sauvignon’ (*Vitis vinifera* L.) were rooted in pots containing a mixture of 4 peat moss: 6 perlite (V/V) and grown in a greenhouse under mist conditions. When the cuttings were rooted, they were repotted into larger pots, and grown for about 10 weeks in a greenhouse at 70–80% relative humidity. During the daytime, temperature was at most 25°C and the maximum photosynthetic active radiation (PAR) was about 1,000 μmol m^-2^ s^-1^. During night time, the temperature was maintained above 18°C. Young grapevines with identical growth (10 leaves) were acclimated for two days in a controlled environment room (70 - 80% relative humidity, 25/18°C day/night cycle and PAR at 800 μmol m^-2^ s^-1^) and divided into two groups. On the following day (the first day of the experiment, Day 1), one group of grapevines was kept at 25/18°C day/night in the controlled environment room as the control. The other group was treated at 45°C in another controlled environment room (except for temperature, the other conditions were the same as the control) from 9:00 am to 14:30 pm. The stressed grapevines were then allowed to recover at 25°C rapidly (from 45°C to 25°C for at about 15 min). Then all conditions were the same as the control until 9:30 am on day 2. Leaf samples of the treatment and control were taken at 14:30 pm on Day 1 and 9:00 am on day 2 for transcriptomic analyses. Three independently replicated experiments were executed.

### RNA extraction, amplification, labeling and hybridization

Total RNA were extracted from grape leaves using Trizol reagent (Invitrogen, Carlsbad, CA) according to the manufacturer’s instructions, and digested with DNase I at 37°C for 15 min to remove any contaminating DNA. The RNA was cleaned up with RNeasy Kit (Qiagen, Hilden, Germany) and the quantities and qualities were determined by spectrophotometry and 1% formaldehyde denaturing gel electrophoresis. The samples with bright bands of ribosomal 28S to 18S RNA in a ratio >1.5:1 were used for microarray analysis. The Affymetrix GeneChip *Vitis vinifera* (Grape) Genome Array, which contains 15,700 probe sets to cover 14,000 *V. vinifera* transcripts and 1,700 transcripts from other *Vitis* species, was used for microarray analysis. Hybridization, data capture, and analysis were performed by CapitalBio Corporation (Beijing, China), a service provider authorized by Affymetrix Inc. (Santa Clara, CA). Briefly, 200 ng of total RNA was used for cDNA synthesis, and produce biotin-tagged cRNA with MessageAmpTM Premier RNA Amplification Kit (Ambion). A 10 μg fragmented cRNA, with control oligo B2 and eukaryotic hybridization controls (bioB, bioC, bioD, cre), was hybridized to each GeneChip array at 45°C for 16 h (Affymetrix Gene Chip Hybridization Oven 640) according to the manufacturer’s instructions. After hybridization, the GeneChip arrays were washed, and then stained with streptavidin phycoerythrinonan (SAPE) with an Affymetrix Fluidics Station 450 followed by scanning with an Affymetrix GeneChip Scanner 3000 7 G.

### Microarray data processing

Microarray image files (CEL) were loaded into a DNA-Chip Analyzer (dChip) software package [80]. The model-based expression values were calculated in dChip, followed by normalization via the program default method (Invariant Set Normalization). An unpaired two-group comparison for all probe sets was performed. Genes were determined to have altered expression levels in the treated samples versus control samples based on the following criteria: (1) P-value < 0.001; (2) Fold change Treatment/Control >2 or <0.5. The lower confidence bound (LCB) of the 90% confidence interval of the fold changes was used [81]; (3) An absolute difference between the means of the expression levels of the two groups was greater than 50; (4) Present calls in both samples are larger than 20%. The reliability of the comparison criteria was assessed by checking the FDR (False Discovery Rate) when permuting samples 200 times. Genes that satisfied all of the above criteria were chosen for further analysis. The gene annotation and functional categories were performed using data at PLEXdb ( http://www.plexdb.org/) and based upon Deluc et al. [82]. All data have been submitted to the PLEXdb, and the accession number is VV40.

### qRT-PCR

Total RNA extraction was the same as that used for microarray analysis described above. The total RNA was treated with DNase I (Promega) to avoid DNA contamination. One microgram of RNA was reverse transcribed using the Superscript II reverse transcriptase (Invitrogen) with an oligo(dT)_15_ primer according to the manufacturer’s instructions (Tiangen Biotech, Beijing, China). qRT-PCR experiments were conducted using Real Master Mix (SYBR Green) (Tiangen Biotech, Beijing, China). Reactions were carried out on a MX 3000 multicolor real-time detection system. The following standard thermal profile was used for all PCR experiments: 94°C for 2 min; 40 cycles of 95°C for 15 s, 56°C for 18 s and 68°C for 20 s. Fluorescence signals were captured at the end of each cycle, and the melting curve analysis was performed from 68°C to 95°C. Gene-specific primers were designed using the Primer5 software (see Additional file 1). The amplification of 18S rRNA gene sequence (GQ849399) was used as the internal control to normalize all the data [83,84]. Analyses of qRT-PCR data used the classic (1 + E)^-ΔΔCT^ method (C_T_ is the threshold cycles of one gene, E is the amplification efficiency). ΔC_T_ is equal to the difference in threshold cycles for target (X) and reference (R) (C_T,X_-C_T,R_), while the ΔΔC_T_ is equal to the difference of ΔC_T_ for control (C) and treatment (T) (ΔC_T,T_-ΔC_T,C_). The amplification system (e.g., primer and template concentrations) was properly optimized, and the efficiency was close to 1. So the amount of target, normalized to an endogenous reference and relative to a calibrator, is given by:

Amount of target =2^-ΔΔCT^.

## Competing interests

The authors declare that they have no competing interests.

## Authors’ contributions

GTL, LJW, SHL and JFW carried out the genomic experiments, or conducted the study design and data analysis. WD, BHW, HGX, PGF participated in the study design and in data analysis. LJW, GTL, SHL, GC and ZWD drafted the manuscript. All authors read, revised and approved the final manuscript.

## Supplementary Material

Additional file 1 Gene-specific primers for qRT-PCR. Click here for file

Additional file 2 **Linear correlation analysis (r = 0.982) between qRT-PCR and microarray results for 12 genes.** X; log_2_ fold change value from microarray data; Y: log_2_ fold change value from qRT-PCR data. Click here for file

Additional file 3 Genes upregulated during heat stress (HS) and downregulated after the subsequent recovery (RC) in grape leaves. Click here for file

Additional file 4 Genes upregulated during heat stress (HS) and after the subsequent recovery (RC) in grape leaves. Click here for file

Additional file 5 Genes downregulated during heat stress (HS) and upregulated after the subsequent recovery (RC) in grape leaves. Click here for file

Additional file 6 Genes downregulated during heat stress and after the subsequent recovery in grape leaves. Click here for file

Additional file 7 Genes upregulated or downregulated unique to heat stress in grape leaves. Click here for file

Additional file 8 Genes upregulated or downregulated unique to the recovery in grapevine leaves. Click here for file
