# Supplementary material for: Transcriptomic analysis of grape (Vitis vinifera L.) leaves during and after recovery from heat stress
Source: BMC Plant Biol. 2012 Sep 28;12:174. doi: 10.1186/1471-2229-12-174 (PMC3497578; doi:10.1186/1471-2229-12-174)
Supplement: Additional file 1 — Gene-specific primers for qRT-PCR. [file 1471-2229-12-174-S1.doc]

Additional file 1 Gene-specific primers for RT-PCR

| Probe set | Gene name description |  | Primer |
| --- | --- | --- | --- |
| 1615503_at | Hsp101 | F | CACACCAAGCCAAACCATACAC |
|  |  | R | CGAAGAAATAGAAGACGAAGAGGAT |
| 1608828_at | Hsp25.7 | F | GAGGGCAGGCAAACATAATC |
|  |  | R | CCACTGTAGGCAAGAGGGTC |
| 1612385_at | Hsp17. | F | GGAGAATGTGAAGGTGGAGGA |
|  |  | R | CTTGACACTGACATAGCCGAAAT |
| 1614330_at | Hsp19 | F | CCTCTCTCCGCTGATCACCA |
|  |  | R | GTACCCCAATCCCTACGCCT |
| 1622628_at | Hsp16.1 | F | GGAGGAAGGGTGCAAATACG |
|  |  | R | CAGTCAAAACCCCATTCTCACAT |
| 1613858_at | Hsp22 | F | CTCCGCCCACATCATCACT |
|  |  | R | CCTCAACACCCCATTCTCAAG |
| 1609838_at | Hsp15.7 | F | CATCCGTGGCTTTCCTTTC |
|  |  | R | CACCTTTATGTCGTCTTTGCTC |
| 1610122_at | HSF30 | F | CATCAAAGGATACAAAAGAACAGG |
|  |  | R | TCAGGTCCAAATCATGAAGAAAT |
| 1611710_at | Class IV chitinase | F | CGTATTCTGGGTTCGGCA |
|  |  | R | TGACCGGAGACACATGGATAT |
| 1610011_s_at | PR10 | F | CTTAACGGGCTCTTCACTGACC |
|  |  | R | GCTCAAAGTGGTGCCTTCTCC |
| 1616889_at | HSP23.6 | F | CGAGTAAAAGTTTCATCGGGTT |
|  |  | R | ACGACAAGCATCCAAACAGAC |
| 1608762_at | HSF7 | F | CGAACTCATCACCCGCCA |
|  |  | R | TCCGTCAACAAGTCCAGCG |
| reference gene | 18S | F | GATTTCGGTCCTATTCTGTTGG |
|  |  | R | CTTTCGCAGTTGTTCGTCTTTC |
