# Supplementary material for: Transcriptomic analysis of grape (Vitis vinifera L.) leaves during and after recovery from heat stress
Source: BMC Plant Biol. 2012 Sep 28;12:174. doi: 10.1186/1471-2229-12-174 (PMC3497578; doi:10.1186/1471-2229-12-174)
Supplement: Additional file 2 — Linear correlation analysis (r = 0.982) between qRT-PCR and microarray results for 12 genes. X; log2 fold change value from microarray data; Y: log2 fold change value from qRT-PCR data. [file 1471-2229-12-174-S2.docx]

Additional file 2 Linear regression analysis between qRT-PCR and microarray results (r = 0.982) for 12 genes.


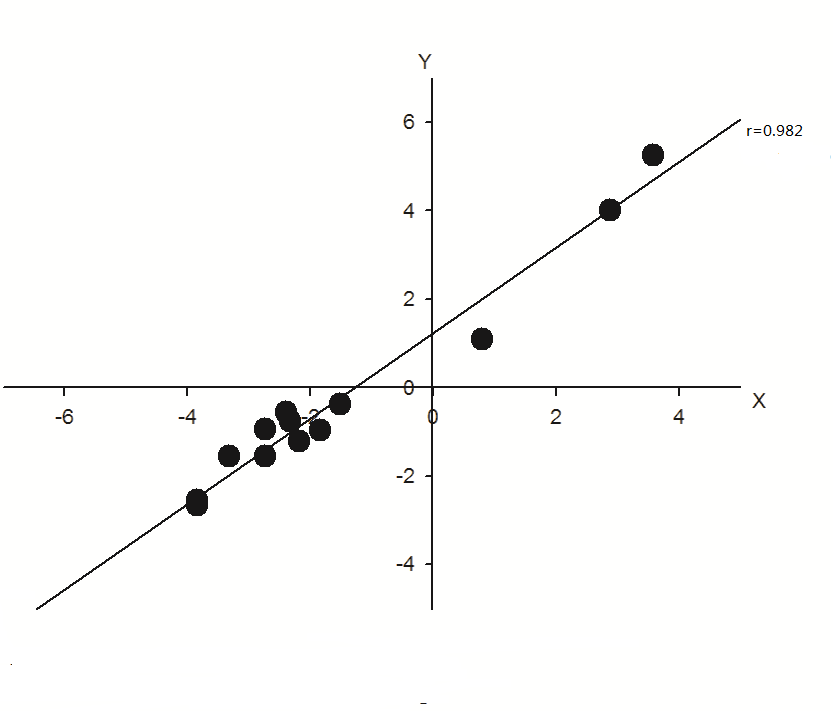


Note: The values of X-axis represent the fold change value of Microarray after log_2_ while the values of Y-axis represent the fold change values of RT-PCR after log_2_.
