# Supplementary material for: Transcriptomic analysis of grape (Vitis vinifera L.) leaves during and after recovery from heat stress
Source: BMC Plant Biol. 2012 Sep 28;12:174. doi: 10.1186/1471-2229-12-174 (PMC3497578; doi:10.1186/1471-2229-12-174)
Supplement: Additional file 3 — Genes upregulated during heat stress (HS) and downregulated after the subsequent recovery (RC) in grape leaves. [file 1471-2229-12-174-S3.docx]

**Additional file 3 Genes upregulated during heat stress (HS) and downregulated after the subsequent recovery (RC) in grape leaves**

| **Category** | **Probe sets** | **Accession** | **Fold change** | | **Gene name description** |
| --- | --- | --- | --- | --- | --- |
|  |  |  | **Up-regulated by HS** | **Down-regulated by RC** |  |
| Cell rescue | 1611643_at | CF519104 | 2.22 | 0.14 | Hin1 like protein |
|  | 1618545_a_at | CB346750 | 2.28 | 0.28 | Stress-induced protein sti1 |
|  | 1609808_at | CB349851 | 40.98 | 0.31 | Galactinol synthase |
|  | 1621902_at | CB345928 | 51.77 | 0.31 | Galactinol synthase |
| Prtotein fate | 1619616_at | BQ798767 | 2.32 | 0.35 | HSP18.6 |
|  | 1614968_at | CB914034 | 4.04 | 0.22 | HSP62.6 |
|  | 1611195_at | CB914034 | 4.78 | 0.19 | HSP40 |
|  | 1616067_at | CF415216 | 4.87 | 0.33 | CPN10 |
|  | 1616369_at | CF371742 | 5.13 | 0.31 | HSP70 |
|  | 1621357_s_at | CF518673 | 5.75 | 0.13 | HSC71.5 |
|  | 1609838_at | BM436450 | 7.39 | 0.20 | HSP15.7 |
|  | 1616811_at | CB347142 | 8.87 | 0.15 | HSP37.1 |
|  | 1615503_at | CF208342 | 9.39 | 0.15 | HSP101 |
|  | 1622628_at | CB349013 | 9.58 | 0.07 | HSP16.1 |
|  | 1610122_at | CF517531 | 10.99 | 0.07 | HSF30 |
|  | 1622489_at | BM436450 | 11.04 | 0.17 | HSP15.7 |
|  | 1620960_a_at | CF515315 | 11.06 | 0.35 | HSP18.3 |
|  | 1616889_at | CB348538 | 12.01 | 0.10 | HSP22 |
|  | 1609554_at | BQ794473 | 19.43 | 0.05 | HSP18.3 |
|  | 1618391_at | CD719685 | 20.32 | 0.04 | HSP17.5 |
|  | 1612385_at | CB350287 | 23.49 | 0.22 | HSP17.6 |
|  | 1616145_a_at | BQ794473 | 25.48 | 0.08 | HSP18.3 |
|  | 1608828_at | CF519164 | 27.00 | 0.10 | HSP25.7 |
|  | 1614330_at | CB343702 | 30.99 | 0.15 | HSP11.1 |
|  | 1620348_at | CB347135 | 43.15 | 0.07 | HSP22 |
|  | 1607291_at | CB007647 | 85.71 | 0.06 | HSP80.1 |
|  | 1610032_at | CF405330 | 101.39 | 0.01 | HSP17.5 |
|  | 1611192_at | CB348409 | 126.05 | 0.16 | HSP17.5 |
|  | 1618066_a_at | CF515990 | 159.96 | 0.04 | HSP80.1 |
|  | 1613858_at | CB350012 | 374.20 | 0.35 | HSP22 |
| Metabolism | 1614487_at | CF403809 | 2.39 | 0.45 | 3-hydroxy-3-methylglutaryl coenzyme A |
|  | 1612435_s_at | CF404469 | 3.32 | 0.24 | Pyridoxin biosynthesis PDX1-like protein |
|  | 1612404_at | CD717201 | 3.32 | 0.28 | Pyridoxin biosynthesis protein ER1 |
| Transcription | 1621552_at | BM437510 | 2.63 | 0.32 | Ethylene-responsive transcriptional coactivator-like protein |
| Signal transduction | 1608981_at | CF209511 | 2.66 | 0.30 | Phospholipase |
|  | 1618587_at | CF518131 | 5.12 | 0.17 | Regulator of gene silencing |
|  | 1616487_at | CA811061 | 5.13 | 0.15 | Regulator of gene silencing |
|  | 1622014_at | CF404401 | 7.86 | 0.08 | Regulator of gene silencing |
| Development | 1606746_a_at | BQ798562 | 15.63 | 0.24 | Ripening regulated protein |
| Storage protein | 1619225_s_at | CA809011 | 2.96 | 0.40 | Seed maturation protein PM37 |
