# Supplementary material for: Transcriptomic analysis of grape (Vitis vinifera L.) leaves during and after recovery from heat stress
Source: BMC Plant Biol. 2012 Sep 28;12:174. doi: 10.1186/1471-2229-12-174 (PMC3497578; doi:10.1186/1471-2229-12-174)
Supplement: Additional file 4 — Genes upregulated during heat stress (HS) and after the subsequent recovery (RC) in grape leaves. [file 1471-2229-12-174-S4.docx]

**Additional file 4 Genes upregulated during heat stress (HS) and after the subsquent recovery (RC) in grape leaves**

| **Category** | **Probe sets** | **Accession** | **Fold change** | | **Gene name description** |
| --- | --- | --- | --- | --- | --- |
|  |  |  | **Up-regulated by HS** | **Up-regulated by RC** |  |
| Cell rescue | 1613067_at | CB968801 | 2.66 | 2.83 | RD22-like protein |
|  | 1609901_at | CF212785 | 4.39 | 15.64 | Monothiol glutaredoxin |
| Protein fate | 1621652_at | CF518056 | 7.50 | 3.96 | HSP20 |
| Metabolism | 1619383_s_at | BQ794831 | 3.17 | 4.10 | Beta-D-galactosidase |
|  | 1606449_at | CF210281 | 3.26 | 4.08 | 1-deoxy-D-xylulose 5-phosphate |
|  | 1617693_at | CB920839 | 3.32 | 3.37 | Omega-6 fatty acid desaturase |
|  | 1606863_at | CD714568 | 4.06 | 3.51 | Ripening-related protein-like |
|  | 1622687_at | CB344274 | 4.13 | 2.76 | Cytochrome P450 |
| Transcription | 1621440_at | CD004030 | 3.48 | 2.82 | g27 (Transcription regulator protein) |
| Interaction with environment | 1606517_at | CB347191 | 2.77 | 3.88 | Putative thaumatin-like protein |
