# Supplementary material for: Transcriptomic analysis of grape (Vitis vinifera L.) leaves during and after recovery from heat stress
Source: BMC Plant Biol. 2012 Sep 28;12:174. doi: 10.1186/1471-2229-12-174 (PMC3497578; doi:10.1186/1471-2229-12-174)
Supplement: Additional file 5 — Genes downregulated during heat stress (HS) and upregulated after the subsequent recovery (RC) in grape leaves. [file 1471-2229-12-174-S5.docx]

**Additional file 5 Genes downregulated during heat stress (HS) and upregulated after the subsequent recovery (RC) in grape leaves**

| **Category** | **Probe sets** | **Accession** | **Fold change** | | **Gene name description** |
| --- | --- | --- | --- | --- | --- |
|  |  |  | **Down-regulated by HS** | **Up-regulated by RC** |  |
| Cell rescue | 1608586_at | CF514688 | 0.30 | 3.27 | Peroxidase 42 |
|  | 1607967_at | CB009400 | 0.42 | 4.43 | Alcohol dehydrogenase 7 |
| Prtotein fate | 1615659_at | CF373840 | 0.35 | 2.27 | F6F3.10 protein |
|  | 1612873_at | CF215155 | 0.36 | 2.16 | Subtilisin proteinase-like |
| Metabolism | 1612710_at | CF415064 | 0.02 | 16.38 | Acid phosphatase |
|  | 1611542_at | CB971080 | 0.14 | 5.15 | Polyphenol oxidase |
|  | 1610935_at | CF404728 | 0.15 | 2.60 | Cytochrome P450 |
|  | 1619522_at | AY043231.1 | 0.16 | 3.13 | Putative beta-galactosidase BG1 |
|  | 1607426_at | AF159124.1 | 0.22 | 4.39 | Beta-galactosidase |
|  | 1622295_at | CF215662 | 0.24 | 4.11 | Beta-D-galactosidase |
|  | 1608393_at | CF403620 | 0.42 | 2.57 | ADP-glucose pyrophosphorylase |
| Signal transduction | 1622444_at | CF212366 | 0.34 | 2.60 | F14J22.4 protein |
| Transport regulation | 1617656_at | CF513562 | 0.22 | 2.61 | Pom14 protein |
|  | 1611312_s_at | BQ796848 | 0.39 | 3.21 | Aquaporin |
|  | 1622157_at | CF404210 | 0.40 | 3.11 | Triose phosphate/phosphate |
|  | 1610603_at | AF271660.1 | 0.42 | 2.85 | Aquaporin |
| Engery | 1620504_at | CB342755 | 0.44 | 2.15 | Putative cytochrome c |
| Cell fate | 1617150_at | BQ792231 | 0.17 | 2.44 | Expansin |
| Biogenesis of cellular components | 1619147_at | BQ796918 | 0.15 | 2.42 | Putative proline-rich cell |
