# Supplementary material for: Transcriptomic analysis of grape (Vitis vinifera L.) leaves during and after recovery from heat stress
Source: BMC Plant Biol. 2012 Sep 28;12:174. doi: 10.1186/1471-2229-12-174 (PMC3497578; doi:10.1186/1471-2229-12-174)
Supplement: Additional file 6 — Genes downregulated during heat stress and after the subsequent recovery in grape leaves. [file 1471-2229-12-174-S6.docx]

**Additional file 6 Genes downregulated during heat stress (HS) and after the subsequent recovery (RC) in grape leaves**

| **Category** | **Probe sets** | **Accession** | **Fold change** | | **Gene name description** |
| --- | --- | --- | --- | --- | --- |
|  |  |  | **down-regulated**  **by HS** | **down-regulated by RC** |  |
| Cell rescue | 1613461_s_at | AF532966.1 | 0.03 | 0.19 | Class IV chitinase |
|  | 1611710_at | AF532966.1 | 0.04 | 0.19 | Class IV chitinase |
|  | 1610011_s_at | CF200913.1 | 0.06 | 0.28 | Pathogenesis-related protein 10 |
|  | 1610704_at | CA809376 | 0.12 | 0.15 | Pathogenesis-related protein 10 |
|  | 1611117_at | CF201368.1 | 0.12 | 0.26 | Dirigent protein |
|  | 1618835_s_at | BQ797163 | 0.14 | 0.12 | PR-4 type protein |
|  | 1620518_at | CF201341.1 | 0.16 | 0.19 | PR-4 type protein |
|  | 1618561_at | CF204500.1 | 0.32 | 0.22 | Short-chain alcohol |
|  | 1613999_x_at | CF202364.1 | 0.39 | 0.28 | Chitinase III |
|  | 1621431_at | CF205258.1 | 0.40 | 0.26 | Peroxidase precursor |
|  | 1608864_s_at | CF202364.1 | 0.40 | 0.27 | Chitinase III |
|  | 1621244_s_at | CD797552 | 0.41 | 0.30 | Short-chain alcohol dehydrogenase |
| Protein fate | 1610524_s_at | CD798339 | 0.34 | 0.25 | HSP70-2 |
|  | 1611445_at | BQ796039 | 0.42 | 0.46 | Protein kinase-like protein |
|  | 1613630_s_at | BQ794547 | 0.45 | 0.41 | HSP71 |
| Metabolism | 1606750_at | AY059638.1 | 0.04 | 0.14 | Stilbene synthase |
|  | 1608009_s_at | S63221.1 | 0.06 | 0.13 | Stilbene synthase 2 |
|  | 1619916_s_at | CD715143 | 0.09 | 0.09 | Beta-1,3-glucanase |
|  | 1607713_s_at | AF487826.1 | 0.16 | 0.17 | Putative serine hydrolase |
|  | 1612124_at | AF239740.1 | 0.20 | 0.46 | Caffeic acid O-methyltransferase |
|  | 1609744_at | CF203760.1 | 0.22 | 0.44 | Putative alanine acetyl transferase |
|  | 1621688_at | CF415096 | 0.22 | 0.11 | Glycerophosphoryl diester |
|  | 1622369_at | CB342790 | 0.24 | 0.38 | Germin-like protein |
|  | 1612393_s_at | CF207031.1 | 0.27 | 0.42 | T12C24.9 |
|  | 1615401_at | CB342555 | 0.28 | 0.22 | Flavonol 3-O-glucosyltransferase 2 |
|  | 1616188_at | CD799282 | 0.33 | 0.41 | Putative esterase |
|  | 1617124_at | CD715446 | 0.40 | 0.42 | Beta-amylase PCT-BMYI |
|  | 1610722_at | BQ797980 | 0.44 | 0.45 | Beta-1,3-glucanase |
| Transcription | 1609636_at | CD006434 | 0.16 | 0.37 | SPF1 protein |
|  | 1620319_s_at | CF205531.1 | 0.19 | 0.41 | Myb-related transcription factor |
|  | 1618260_s_at | CD799434 | 0.19 | 0.31 | Myb-related transcription factor |
|  | 1621876_at | BQ798834 | 0.20 | 0.18 | NAC domain protein NAC1 |
|  | 1610775_s_at | CF206474.1 | 0.31 | 0.33 | WRKY transcription factor-b |
|  | 1614806_s_at | CB343751 | 0.37 | 0.43 | Putative WRKY4 transcription factor |
|  | 1611583_at | CF405989 | 0.40 | 0.44 | Similarity to AP2 domain |
| Signal transduction | 1612734_at | CD717076 | 0.10 | 0.33 | Receptor kinase-like protein |
|  | 1609131_at | CF514932 | 0.21 | 0.43 | T1N24.22 protein |
|  | 1610522_a_at | CD797296 | 0.31 | 0.44 | Putative leucine-rich repeat |
|  | 1619208_at | CA814525 | 0.41 | 0.24 | Hypothetical protein |
| Transport regulation | 1621817_at | CB978007 | 0.09 | 0.42 | T13D8.29 protein |
|  | 1611820_at | CB914713 | 0.13 | 0.38 | T13D8.29 protein |
|  | 1610394_at | CA816120 | 0.14 | 0.05 | Ferritin |
|  | 1622455_at | CF207039.1 | 0.31 | 0.45 | Putative peptide transporter |
| Engery | 1617922_at | CF405309 | 0.36 | 0.44 | Lipoxygenase |
|  | 1607193_at | BQ796845 | 0.37 | 0.26 | Alternative oxidase |
| Interaction with environment | 1615458_at | CB969727 | 0.03 | 0.29 | TO118-23rc |
|  | 1622745_at | BQ796736 | 0.09 | 0.13 | Putative quinone oxidoreductase |
|  | 1620390_s_at | AF532965.1 | 0.17 | 0.18 | Thaumatin-like protein |
|  | 1610989_at | CA811091 | 0.21 | 0.38 | Probable glutathione S-transferase |
|  | 1610880_s_at | CF371851 | 0.22 | 0.31 | Auxin and ethylene responsive  GH3-like protein |
|  | 1622360_at | CB981283 | 0.29 | 0.29 | Nitrilase 4B |
| Protein with binding function | 1620207_at | CF213689 | 0.23 | 0.26 | Yippee-like protein |
| Biogenesis of cellular components | 1616822_at | AF220196.1 | 0.23 | 0.44 | Proline-rich protein 1 |
