# Supplementary material for: Transcriptomic analysis of grape (Vitis vinifera L.) leaves during and after recovery from heat stress
Source: BMC Plant Biol. 2012 Sep 28;12:174. doi: 10.1186/1471-2229-12-174 (PMC3497578; doi:10.1186/1471-2229-12-174)
Supplement: Additional file 7 — Genes upregulated or downregulated unique to heat stress in grape leaves. [file 1471-2229-12-174-S7.docx]

**Additional file 7 Genes upregulated or downregulated unique to heat stress in grape leaves**

| **Category** | **Probe sets** | **Accession** | **Fold change** | | **Gene name description** |
| --- | --- | --- | --- | --- | --- |
| Cell rescue | 1616133_at | CB340026 | 7.59 | Major latex protein | |
|  | 1618137_at | CB969885 | 5.21 | Cytosolic ascorbate peroxidase (APX) | |
|  | 1611871_at | CF415063 | 3.18 | Dehydroascorbate reductase(DHAR) | |
|  | 1606514_at | CD799006 | 2.73 | Putative wound-induced protein | |
|  | 1615226_at | CF205569.1 | 2.48 | Expressed protein | |
|  | 1619756_at | CB003378 | 2.48 | Glutaredoxin-like protein | |
|  | 1610593_at | CD715465 | 2.44 | Putative salt-inducible protein | |
|  | 1606772_at | CF606865 | 2.26 | Putative salt-inducible protein | |
|  | 1619178_at | CB349106 | 2.21 | Putative ER6 protein | |
|  | 1618164_at | CD800719 | 2.11 | Putative cytochrome P450 | |
|  | 1618491_s_at | CB974861 | 0.46 | Similarity to endo-1 | |
|  | 1610504_at | CB974454 | 0.44 | Putative elicitor-responsive gene-3 | |
|  | 1609780_at | CA810742 | 0.44 | Putative ER6 protein | |
|  | 1615301_at | CF373109 | 0.44 | Avr9/Cf-9 rapidly elicited protein 76 (Fragment) | |
|  | 1614972_at | CB969853 | 0.43 | Short-chain dehydrogenase Tic32 | |
|  | 1620436_a_at | CB910355 | 0.43 | Putative low-temperature induced protein | |
|  | 1611890_at | BQ797015 | 0.42 | Glutathione S-transferase GST 14 | |
|  | 1607645_at | CA817594 | 0.42 | Pathogen-related protein | |
|  | 1606987_at | CF405059 | 0.40 | Probable disease resistance protein | |
|  | 1608159_at | CB969766 | 0.39 | Short-chain dehydrogenase Tic32 | |
|  | 1613132_s_at | CK136916.1 | 0.38 | Secretory peroxidase | |
|  | 1611400_at | CF510785 | 0.37 | Cold shock protein-1 | |
|  | 1616703_at | BQ800335 | 0.36 | F3M18.8 (At1g28480) | |
|  | 1609321_at | CA817974 | 0.32 | Anionic peroxidase precursor | |
|  | 1620505_at | Z54234.1 | 0.32 | Basic endochitinase precursor | |
|  | 1617308_at | CF568997 | 0.31 | Putative dirigent protein | |
|  | 1614259_at | CB968937 | 0.27 | Putative stress related chitinase | |
|  | 1617430_s_at | Z54234.1 | 0.26 | Basic endochitinase precursor | |
|  | 1612108_at | BQ800036 | 0.25 | Avr9/Cf-9 rapidly elicited protein 146 | |
|  | 1619115_s_at | CA817457 | 0.21 | Disease resistance response protein-like | |
|  | 1618775_at | BM436456 | 0.21 | Putative pathogenesis-related protein | |
|  | 1621829_at | CF608402 | 0.20 | Erg-1 | |
|  | 1616495_at | CF517304 | 0.20 | Glutathione S-transferase GST 24 | |
|  | 1614862_at | BQ797749 | 0.20 | Putative short chain alcohol dehydrogenase | |
|  | 1607127_s_at | CA809554 | 0.19 | Putative pathogenesis related protein | |
|  | 1608262_at | AF053341.1 | 0.15 | Chitinase precursor | |
|  | 1615967_at | CF211449 | 0.14 | Peroxidase 73 precursor | |
|  | 1620137_s_at | CB911784 | 0.14 | Seed-specific low molecular weight sulfur-rich protein | |
|  | 1606845_at | CF212991 | 0.13 | Erg-1 | |
|  | 1616735_at | CF604749 | 0.13 | Expressed protein | |
|  | 1615552_at | CA807930 | 0.07 | Galactinol synthase | |
|  | 1616064_at | CF205270.1 | 0.06 | Class IV endochitinase | |
|  | 1621319_s_at | CB981122 | 0.04 | Class IV chitinase | |
|  | 1607557_at | CF202548.1 | 0.03 | Class IV chitinase | |
| Prtotein fate | 1620985_at | CB348258 | 43.00 | HSP21 | |
|  | 1621709_at | CF518506 | 10.87 | Hsp70 | |
|  | 1614448_at | CB976275 | 4.59 | ClpB | |
|  | 1607070_at | CB347331 | 3.82 | ClpB3 | |
|  | 1619626_at | CB005796 | 3.23 | E3 ubiquitin-protein ligase ATL23 | |
|  | 1619819_at | CF207778 | 3.07 | HSP89.1 | |
|  | 1618693_at | CB348122 | 2.59 | Chaperonin-60 beta subunit precursor | |
|  | 1621565_s_at | CF516181 | 2.59 | Thioredoxin family protein | |
|  | 1615777_at | CD719431 | 2.45 | Putative prolyl endopeptidase | |
|  | 1615731_at | CD801373 | 2.40 | HSP83-like | |
|  | 1619547_at | CD719431 | 2.28 | Putative prolyl endopeptidase | |
|  | 1609491_at | CA809519 | 2.28 | Putative protease | |
|  | 1614692_at | CF518918 | 2.19 | HSP88.1 | |
|  | 1609502_at | CF372687 | 2.17 | CPN60-2 | |
|  | 1613057_at | CD711210 | 2.12 | HSP88.1 | |
|  | 1608213_at | CF209965 | 0.46 | Subtilisin-like protease (Fragment) | |
|  | 1609576_at | BQ794740 | 0.45 | BTH-induced protein phosphatase 2C 2 K2 form | |
|  | 1621391_at | CD801716 | 0.44 | Mevalonate kinase | |
|  | 1621854_at | CF204105.1 | 0.43 | T28P6.6 protein | |
|  | 1617431_at | CB344842 | 0.42 | Putative subtilisin-like serine protease AIR3 | |
|  | 1606621_at | CF513895 | 0.40 | Serine/threonine-protein kinase | |
|  | 1611674_at | BQ792639 | 0.39 | Ring H2 zinc finger | |
|  | 1607752_at | CF513611 | 0.35 | Hypothetical protein (Fragment) | |
|  | 1620289_at | CF510550 | 0.35 | Papain-like cysteine proteinase isoform I | |
|  | 1621666_at | CF404585 | 0.34 | Putativepod-specific dehydrogenase SAC25 | |
|  | 1620442_at | CB971589 | 0.34 | Putative serine carboxypeptidase II | |
|  | 1613980_at | CB968799 | 0.33 | Subtilisin-like serine protease | |
|  | 1616531_at | CF405878 | 0.30 | Protein disulfide isomerase (PDI)-like protein 3 | |
|  | 1611080_at | CF372804 | 0.30 | DnaJ-like protein | |
|  | 1613265_s_at | CB972047 | 0.29 | Tyrosine specific protein phosphatase family protein | |
|  | 1609665_a_at | CB005515 | 0.28 | Probable protein phosphatase 2C 34 | |
|  | 1611873_s_at | CD004402 | 0.28 | Zinc finger (C3HC4-type RING finger)-like | |
|  | 1609079_at | BQ796278 | 0.28 | Putative pyruvate kinase | |
|  | 1618638_at | CD003864 | 0.21 | Protein kinase 2 | |
|  | 1607369_at | CF213621 | 0.21 | T2P11_13 | |
|  | 1618022_at | BM437052 | 0.11 | Cysteine protease | |
| Metabolism | 1611095_a_at | CB348001 | 5.72 | DC1.2 homologue | |
|  | 1612763_at | CF373209 | 5.19 | Putative ripening-related protein | |
|  | 1608206_at | CA813760 | 4.15 | Imidazole glycerol phosphate synthase hisHF | |
|  | 1615421_at | CF516532 | 3.18 | T1K7.26 protein | |
|  | 1607221_at | CF405062 | 3.02 | Strictosidine synthase family protein | |
|  | 1614668_at | CF373733 | 2.95 | Putative adenine phosphoribosyl transferase | |
|  | 1609390_at | CD798340 | 2.91 | Putative sterol 4-alpha-methyl-oxidase | |
|  | 1622535_at | CB976419 | 2.78 | Putative ripening-related protein | |
|  | 1619335_at | CB005450 | 2.75 | Putative ripening-related protein | |
|  | 1620325_at | CF512180 | 2.70 | Homocysteine S-methyltransferase 2 | |
|  | 1611508_at | CF514821 | 2.47 | Thioredoxin family protein | |
|  | 1622591_at | CB981129 | 2.47 | Pectinesterase-like; strong similarity to pollen-specific protein | |
|  | 1617205_at | CF512534 | 2.46 | Tetrahydropicolinate succinylase | |
|  | 1610151_at | CF404642 | 2.44 | Putative protein (belongs to the AAA ATPase family) | |
|  | 1618204_at | BQ795716 | 2.42 | Enoyl-ACP reductase precursor | |
|  | 1616136_at | CF215091 | 2.18 | 5-methyltetrahydropteroyltriglutamate--homocysteine methyltransferase | |
|  | 1607721_at | CB005803 | 0.47 | 3,5-epimerase/4-reductase | |
|  | 1619268_at | CF568884 | 0.47 | Putative aspartate aminotransferase | |
|  | 1614728_at | CF215143 | 0.47 | Syringomycin biosynthesis enzyme-like | |
|  | 1610801_at | CB969609 | 0.46 | Glutamate decarboxylase | |
|  | 1619223_s_at | CB005867 | 0.46 | Sucrose synthase | |
|  | 1606577_at | CB920120 | 0.45 | acid hydroperoxide lyase | |
|  | 1618316_at | CF602990 | 0.45 | Putative glucosyltransferase | |
|  | 1619079_s_at | CB008174 | 0.45 | Cytosolic acetoacetyl-coenzyme A thiolase | |
|  | 1620752_s_at | CA809260 | 0.45 | Reductase 1 | |
|  | 1621305_s_at | CA814305 | 0.45 | Dehydrogenase/reductase SDR family member 4 | |
|  | 1617746_at | CF214456 | 0.45 | 5-methyltetrahydropteroyltriglutamate--homocysteine methyltransferase | |
|  | 1616966_at | CD012505 | 0.45 | Glutamine-fructose-6-phosphate transaminase 2 | |
|  | 1607043_at | BQ793537 | 0.44 | Cytosolic malate dehydrogenase | |
|  | 1617584_s_at | CF211103 | 0.44 | Putative trehalose-6-phosphate synthase/phosphatase | |
|  | 1619986_s_at | CD800813 | 0.43 | UDP-glucose:flavonoid 3-O-glucosyltransferase (Fragment) | |
|  | 1616007_at | CB002013 | 0.43 | Probable galacturonosyltransferase-like 1 | |
|  | 1609520_at | CF210827 | 0.43 | Mannan endo-1,4-beta-mannosidase | |
|  | 1611027_at | CB978747 | 0.43 | Cell wall apoplastic invertase | |
|  | 1620767_at | BQ800601 | 0.43 | Glutamine synthetase | |
|  | 1610427_at | CB978888 | 0.43 | PRE87 protein | |
|  | 1614789_at | BQ799243 | 0.43 | L-asparaginase | |
|  | 1611001_at | CF204537.1 | 0.42 | Beta-1,3-glucanase 1 | |
|  | 1615939_at | BQ799440 | 0.42 | Putative callose synthase 1 catalytic subunit | |
|  | 1614574_at | CF206733.1 | 0.42 | Lysine decarboxylase-like | |
|  | 1621834_at | BQ794561 | 0.42 | Phosphatidic acid phosphatase alpha (EC 3.1.3.4) | |
|  | 1615622_at | CB339248 | 0.41 | PAPS-reductase-like protein precursor | |
|  | 1619011_at | CF208055 | 0.41 | Putative fatty acid elongase 3-ketoacyl-CoA synthase 1 | |
|  | 1610468_at | CF207447 | 0.41 | D-3-phosphoglycerate dehydrogenase | |
|  | 1614355_at | CF604126 | 0.41 | Fumarylacetoacetase | |
|  | 1621005_at | CF213296 | 0.41 | Hydrolase-like protein | |
|  | 1609307_at | CD715818 | 0.41 | 4-coumarate--CoA ligase 1 | |
|  | 1610738_at | CB976949 | 0.41 | Putative lipase | |
|  | 1620891_at | CD715446 | 0.40 | Beta-amylase PCT-BMYI | |
|  | 1606952_at | CB968665 | 0.40 | Putative neutral/alkaline invertase | |
|  | 1607805_s_at | CF605243 | 0.40 | Putative cytochrome P450 | |
|  | 1619357_at | CB972316 | 0.40 | 3-deoxy-D-arabino-heptulosonate 7-phosphate synthase precursor | |
|  | 1611875_at | BQ798070 | 0.40 | Putative gamma-lyase | |
|  | 1615085_at | CF201650.1 | 0.39 | Caffeic acid O-methyltransferase-like protein | |
|  | 1608032_at | CF512550 | 0.39 | Alpha/beta-hydrolase domain-containing protein | |
|  | 1616399_s_at | CD800967 | 0.39 | Arginine decarboxylase | |
|  | 1618777_at | CA809083 | 0.39 | Putatative tyrosine aminotransferase | |
|  | 1614707_at | BQ799313 | 0.39 | Alpha-glucan phosphorylase, H isozyme | |
|  | 1607622_at | CF404670 | 0.38 | 3-hydroxy-3-methylglutaryl-coenzyme A reductase | |
|  | 1621378_at | BQ794342 | 0.38 | Ribose-phosphate pyrophosphokinase 1 | |
|  | 1612571_at | CB036024 | 0.38 | Putative UDP-glucose glucosyltransferase | |
|  | 1616294_s_at | CD802397 | 0.38 | MutT domain protein-like | |
|  | 1618957_at | CF603287 | 0.38 | Os05g0117500 protein | |
|  | 1609712_at | CF205577.1 | 0.38 | Putative cytochrome P450 | |
|  | 1617024_at | CF211973 | 0.38 | 1-deoxy-D-xylulose 5-phosphate synthase | |
|  | 1619983_at | CD802361 | 0.37 | Caffeic acid O-methyltransferase-like protein | |
|  | 1618004_s_at | CB339217 | 0.37 | AlaT1 | |
|  | 1614440_at | BQ796922 | 0.36 | 3-deoxy-D-arabino-heptulosonate 7-phosphate synthase precursor | |
|  | 1614479_at | BQ796528 | 0.36 | Arginine decarboxylase | |
|  | 1622282_at | CD712313 | 0.36 | Putative fructokinase 2 | |
|  | 1610794_at | CB970276 | 0.35 | S-adenosyl-methionine-sterol-C-methyltransferase | |
|  | 1616315_s_at | CB970362 | 0.35 | Putative sterol-C-methyltransferase (Fragment) | |
|  | 1610695_at | CB972676 | 0.35 | Phosphoethanolamine N-methyltransferase | |
|  | 1618893_at | CF204978.1 | 0.35 | Aldo/keto reductase | |
|  | 1609818_at | BQ797366 | 0.34 | Beta-amylase | |
|  | 1614191_s_at | CD802361 | 0.34 | Caffeic acid O-methyltransferase-like protein | |
|  | 1622556_at | CB972747 | 0.34 | Phosphoethanolamine N-methyltransferase 1 | |
|  | 1615614_at | CF405918 | 0.34 | Putative glucosyltransferase (Fragment) | |
|  | 1614153_at | CF207979 | 0.33 | Putative plastidic glucose 6-phosphate dehydrogenase | |
|  | 1614825_at | CF214450 | 0.32 | 3-oxoacyl-[acyl-carrier-protein] reductase | |
|  | 1622451_s_at | CB982889 | 0.32 | S-adenosylmethionine decarboxylase proenzyme | |
|  | 1617683_at | CF604032 | 0.31 | Cuticle protein (Faceless pollen-1) | |
|  | 1619097_a_at | CF213300 | 0.31 | Adenosylhomocysteinase | |
|  | 1620063_at | CB921343 | 0.31 | Beta 1-3 glucanase | |
|  | 1607760_at | CF515831 | 0.30 | Flavonoid 3',5'-hydroxylase 2 | |
|  | 1608741_s_at | CF206716.1 | 0.30 | 1-deoxy-D-xylulose 5-phosphate synthase 1 precursor | |
|  | 1616400_s_at | CF206159.1 | 0.30 | Gamma-glutamylcysteine synthetase | |
|  | 1614423_at | CF517687 | 0.30 | Cinnamoyl CoA reducta | |
|  | 1619682_x_at | CF205002.1 | 0.30 | Caffeic acid O-methyltransferase | |
|  | 1620628_at | CF403671 | 0.30 | Neutral invertase | |
|  | 1616445_at | CD716014 | 0.29 | Putative cinnamoyl CoA reductase | |
|  | 1614923_at | CD004083 | 0.29 | 3-hydroxy-3-methylglutaryl-coenzyme A reductase | |
|  | 1607262_at | CF206222.1 | 0.29 | EDS1-like protein | |
|  | 1614045_at | CF604589 | 0.29 | Aldehyde 5-hydroxylase | |
|  | 1616093_at | CF404665 | 0.28 | Pectinesterase | |
|  | 1609376_at | CB981321 | 0.28 | Ovule/fiber cell elongation protein Ghfe1 | |
|  | 1619450_s_at | CF215109 | 0.28 | Caffeic acid 3-O-methyltransferase 1 | |
|  | 1614428_at | BQ799174 | 0.27 | Gamma-glutamylcysteine synthetase | |
|  | 1608791_at | CB978059 | 0.26 | Flavonol synthase | |
|  | 1615478_at | CB971615 | 0.26 | Aldo-keto reductase | |
|  | 1616434_s_at | AF239740.1 | 0.25 | Caffeic acid O-methyltransferase | |
|  | 1615646_at | CB974276 | 0.25 | Probable methyltransferase PMT26 | |
|  | 1617421_at | CF205705.1 | 0.25 | Isoflavone reductase-like protein 3 | |
|  | 1611872_at | CF404465 | 0.25 | Putative beta-ketoacyl-CoA synthase | |
|  | 1611211_at | CF603196 | 0.25 | 2-dehydro-3-deoxyphosphoheptonate aldolase | |
|  | 1614680_at | CB916405 | 0.25 | Putative 3-isopropylmalate dehydratase large subunit | |
|  | 1621405_at | CF517917 | 0.25 | Plastidic 3-deoxy-D-arabino-heptulosonate 7-phosphate synthase 2 | |
|  | 1622450_at | CB970586 | 0.24 | Putative pectinesterase | |
|  | 1614513_at | CF414215 | 0.24 | carboxylesterase 2 | |
|  | 1609652_s_at | CF215703 | 0.24 | Glucosyltransferase-like protein | |
|  | 1619909_at | CF215233 | 0.23 | adenosylmethionine synthetase 2 | |
|  | 1618096_s_at | BQ794769 | 0.23 | Putative ripening-related protein | |
|  | 1621563_x_at | AF239740.1 | 0.23 | Caffeic acid O-methyltransferase | |
|  | 1611853_at | BQ797611 | 0.23 | HMG-CoA synthase 2 | |
|  | 1614643_at | CF214966 | 0.22 | Caffeoyl-CoA O-methyltransferase | |
|  | 1615974_at | CB915338 | 0.22 | Putative ripening-related protein | |
|  | 1613945_at | CF414185 | 0.22 | Probable beta-D-xylosidase 7 | |
|  | 1614994_at | CB970828 | 0.22 | Putative GDSL-motif lipase/hydrolase | |
|  | 1611613_at | BQ796771 | 0.21 | Vacuolar invertase 1, GIN1 | |
|  | 1615595_at | AF239617.2 | 0.21 | Beta-1,3-glucanase | |
|  | 1607990_at | CB005729 | 0.19 | Enoyl-ACP reductase precursor | |
|  | 1612436_s_at | CF209460 | 0.19 | Isoflavone reductase-like protein 3 | |
|  | 1608252_s_at | BQ797611 | 0.18 | HMG-CoA synthase 2 | |
|  | 1611847_at | BM437829 | 0.18 | Flavonoid 3',5'-hydroxylase | |
|  | 1620590_at | CB005729 | 0.17 | Enoyl-ACP reductase precursor | |
|  | 1610218_s_at | CF215819 | 0.17 | UDP-glucose dehydrogenase | |
|  | 1620905_at | CF215819 | 0.16 | UDP-glucose dehydrogenase | |
|  | 1612836_at | CF403299 | 0.16 | Vacuolar invertase 2, GIN2 | |
|  | 1606429_at | CF373019 | 0.16 | DC1.2 homologue | |
|  | 1616977_at | CD801826 | 0.16 | Putative iron/ascorbate-dependent oxidoreductase | |
|  | 1609506_at | AY043235.1 | 0.15 | Putative cellulase CEL2 | |
|  | 1612560_at | CF510839 | 0.15 | PS60 protein precursor | |
|  | 1612105_at | CF415846 | 0.14 | Putative beta-ketoacyl-CoA synthase | |
|  | 1611601_at | CB977009 | 0.14 | Putative pectinesterase | |
|  | 1622770_at | CF209970 | 0.14 | Cellulase | |
|  | 1622196_at | CF511416 | 0.13 | Homocysteine S-methyltransferase 1 | |
|  | 1612285_at | CF204274.1 | 0.13 | Putative flavanone 3-hydroxylase | |
|  | 1607492_at | CF205018.1 | 0.12 | Putative ripening-related protein | |
|  | 1616158_at | CD801717 | 0.12 | Pectate lyase | |
|  | 1612185_at | CB008768 | 0.12 | Mannan endo-1,4-beta-mannosidase | |
|  | 1608261_at | CF369036 | 0.11 | Putative pectin methylesterase precursor | |
|  | 1618297_s_at | CF208295 | 0.07 | Pectate lyase | |
|  | 1606998_at | CF208295 | 0.07 | Pectate lyase | |
|  | 1618576_at | BQ800615 | 0.05 | Pectate lyase | |
|  | 1610382_at | CF205724.1 | 0.03 | Methyl transferase | |
| Transcription | 1619201_at | CF404561 | 3.62 | TRIPTYCHON (MYB transcription factor) | |
|  | 1617350_at | CB975555 | 2.36 | Lamin | |
|  | 1610804_at | CB918636 | 2.34 | Putative spliceosome associated protein | |
|  | 1610512_at | CD798938 | 2.29 | Myb-related protein | |
|  | 1607451_at | CB345121 | 2.24 | Putative sec14 like protein | |
|  | 1612649_s_at | CA809370 | 0.47 | WRKY transcription factor NtEIG-D48 | |
|  | 1611650_at | CA808599 | 0.46 | WRKY transcription factor NtEIG-D48 | |
|  | 1618700_at | CF609905 | 0.46 | Putative 3'-5' exoribonuclease | |
|  | 1614295_at | BQ792980 | 0.46 | MYC transcription factor (Fragment) | |
|  | 1615011_at | CB346324 | 0.46 | DNA-binding protein | |
|  | 1615524_at | CA810708 | 0.45 | MADS-box protein PTM5 | |
|  | 1620982_at | CB977185 | 0.44 | Squamosa promoter binding protein-like 1 | |
|  | 1616263_s_at | CF212764 | 0.44 | OSJNBa0059E14.21 | |
|  | 1615964_at | CA810315 | 0.43 | NAM / CUC2-like protein | |
|  | 1612039_at | BQ799884 | 0.43 | Putative snRNP splicing factor (At2g03870) | |
|  | 1608936_at | CF214634 | 0.42 | Transcription factor bHLH128 | |
|  | 1619492_at | CK136879.1 | 0.42 | Splicing factor-like protein | |
|  | 1611013_at | CF518927 | 0.41 | Putative zinc finger protein ID1 | |
|  | 1617296_at | CD798287 | 0.41 | GRF1-interacting factor 2 | |
|  | 1608676_s_at | CD798287 | 0.40 | GRF1-interacting factor 2 | |
|  | 1610064_at | CB341653 | 0.39 | SPF1 protein | |
|  | 1606659_s_at | CF210179 | 0.39 | Putative WRKY transcription factor | |
|  | 1609798_at | CB980206 | 0.38 | Trihelix transcription factor GTL2 | |
|  | 1609172_at | BQ792053 | 0.37 | NAC domain protein NAC3 | |
|  | 1621471_s_at | AB073015.1 | 0.36 | Myb-related transcription factor VlMYBD (Fragment) | |
|  | 1619573_at | CD721204 | 0.36 | Zinc-finger protein 1; similar to AZF3 | |
|  | 1617012_at | CD802399 | 0.35 | PPLZ02 protein | |
|  | 1617931_at | CF510771 | 0.34 | Homeobox-leucine zipper protein ATHB-7 | |
|  | 1613804_at | CF514968 | 0.34 | Putative bHLH transcription factor bHLH106 | |
|  | 1609496_at | CB914077 | 0.34 | BHLH transcription factor | |
|  | 1613776_at | BQ796788 | 0.33 | Hypothetical transcription factor | |
|  | 1614953_at | CF519173 | 0.30 | MYB-related protein 306 | |
|  | 1619585_at | CD800299 | 0.28 | ERF-like protein | |
|  | 1617671_s_at | AY395744.1 | 0.28 | Putative ethylene response factor ERF3a | |
|  | 1606975_at | CD799008 | 0.27 | Putative ethylene response factor ERF3a | |
|  | 1622064_at | CF606041 | 0.24 | Myb-related transcription factor VlMYBB1-2 | |
|  | 1619311_at | CB970517 | 0.24 | Pathogenesis-related genes transcriptional activator PTI5 | |
|  | 1617411_at | BQ797398 | 0.23 | Hypothetical protein (Contains bHLH domain) | |
|  | 1613770_s_at | CB971597 | 0.16 | Jasmonate ZIM domain-containing protein 1 | |
|  | 1621784_at | AB073016.1 | 0.16 | Myb-related transcription factor VlMYBB1-2 | |
|  | 1610633_at | BM438112 | 0.16 | Zinc finger protein | |
|  | 1613136_at | CB969589 | 0.07 | Jasmonate ZIM domain-containing protein 7 | |
| Signal transduction | 1611562_at | CF211645 | 4.39 | Strubbelig receptor family 3 | |
|  | 1617924_at | CF516663 | 3.44 | Putative receptor associated protein (Fragment) | |
|  | 1613877_at | BQ800323 | 2.64 | ADP-ribosylation factor-like protein | |
|  | 1609354_at | CB350009 | 2.48 | Putative GTP-binding protein | |
|  | 1614471_at | BQ792249 | 0.46 | Expressed protein | |
|  | 1608006_at | CF517298 | 0.46 | component response regulator-like APRR7 | |
|  | 1608312_at | CA809526 | 0.45 | Rho GDP-dissociation inhibitor 1 (Rho GDI-1) (AtRhoGDI1) | |
|  | 1610295_at | BQ797947 | 0.45 | CBL-interacting protein kinase CIPK25 | |
|  | 1609077_at | CF606671 | 0.43 | 14-3-3 protein | |
|  | 1607367_at | CF203364.1 | 0.43 | Receptor-like kinase | |
|  | 1610660_at | CB008720 | 0.43 | Putative receptor-protein kinase | |
|  | 1618064_at | CF201673.1 | 0.42 | Phospholipase like protein | |
|  | 1612293_at | CB346119 | 0.42 | Serpin-like protein (Fragment) | |
|  | 1612958_at | CF512608 | 0.41 | Protein kinase-like protein | |
|  | 1614595_at | CF512523 | 0.41 | Mitogen-activated protein kinase (Fragment) | |
|  | 1613552_at | CB916656 | 0.41 | Phospholipase C | |
|  | 1607749_at | CF516612 | 0.41 | Putative receptor-like protein kinase | |
|  | 1612132_s_at | CB341731 | 0.41 | Protein phosphatase type 2C, putative | |
|  | 1617710_s_at | CD012500 | 0.41 | Receptor protein kinase-like | |
|  | 1621974_at | CB971118 | 0.40 | MAP kinase phosphatase | |
|  | 1620367_at | CF415391 | 0.40 | Putative receptor-like kinase | |
|  | 1619654_at | CF415234 | 0.40 | Putative phospholipase | |
|  | 1620931_at | CB915389 | 0.39 | Phospholipase C1 | |
|  | 1616189_at | CF511570 | 0.37 | Receptor protein kinase CLAVATA1 precursor | |
|  | 1621727_s_at | CF511469 | 0.37 | Calcium-dependent protein kinase | |
|  | 1611883_at | CB972411 | 0.37 | Serine/threonine protein kinase | |
|  | 1618445_s_at | CF372206 | 0.36 | WAK-like kinase | |
|  | 1619479_a_at | CB980068 | 0.35 | Putative 7-transmembrane G-protein-coupled receptor | |
|  | 1614319_at | CF515648 | 0.35 | Lectin-like receptor kinase 7;3 | |
|  | 1610279_s_at | CF415234 | 0.32 | Putative phospholipase | |
|  | 1615909_s_at | CF518671 | 0.31 | Mitogen-activated protein kinase | |
|  | 1620375_at | CA814054 | 0.31 | GSK-like kinase | |
|  | 1606597_at | CF208068 | 0.30 | F21M12.36 protein | |
|  | 1612200_at | CB971123 | 0.30 | TIP41-like protein | |
|  | 1620797_at | BQ797057 | 0.16 | T1N24.22 protein (Putative receptor protein kinase) | |
| Transport regulation | 1614230_at | CB975652 | 3.72 | Type 1 non-specific lipid transfer protein precursor | |
|  | 1619602_at | CD710774 | 3.46 | Putative mitochondrial dicarboxylate carrier protein | |
|  | 1615843_at | CD710774 | 3.34 | Putative mitochondrial dicarboxylate carrier protein | |
|  | 1612244_s_at | CF512511 | 3.19 | Putative aquaporin PIP2-2 | |
|  | 1612678_at | CF212553 | 2.75 | Putative ABC transporter protein | |
|  | 1613874_at | CB339929 | 2.52 | Protein ycf2 | |
|  | 1607599_at | BM437406 | 2.35 | Putative histidine amino acid transporter | |
|  | 1621190_at | CB341294 | 2.25 | Protein ycf2 | |
|  | 1618682_at | CA814693 | 0.47 | Zinc transporter protein ZIP1 | |
|  | 1614588_s_at | CF406014 | 0.47 | Potential calcium-transporting ATPase 9 | |
|  | 1616767_at | CF213759 | 0.46 | SNAP1 | |
|  | 1611039_s_at | BQ798757 | 0.45 | SNAP1 | |
|  | 1612744_at | CF214403 | 0.45 | ABC transporter | |
|  | 1608136_s_at | CA814693 | 0.44 | Zinc transporter protein ZIP1 | |
|  | 1607681_at | CD802479 | 0.44 | Phosphate transporter | |
|  | 1616229_at | CB344915 | 0.44 | 2-oxoglutarate/malate translocator-like protein | |
|  | 1615540_s_at | BQ796756 | 0.44 | Putative peptide transporter protein | |
|  | 1618243_at | CF604301 | 0.44 | Putative iron inhibited ABC transporter 2 | |
|  | 1616666_at | CF403684 | 0.43 | Mitochondrial carrier protein | |
|  | 1611325_at | CF211447 | 0.43 | ABC transporter | |
|  | 1620486_at | CF207301 | 0.42 | Cation/H+ exchanger (Fragment) | |
|  | 1617470_s_at | BQ797931 | 0.42 | Amino acid permease | |
|  | 1622721_at | CF214391 | 0.41 | Major facilitator protein | |
|  | 1618942_at | CA817511 | 0.41 | Putative phosphate transporter | |
|  | 1618058_at | BQ794484 | 0.40 | Putative anthocyanin permease | |
|  | 1612823_at | CF514127 | 0.40 | Two-pore calcium channel | |
|  | 1614764_at | BQ794515 | 0.39 | Hexose transporter HT2 | |
|  | 1613840_at | BQ800291 | 0.39 | PDR-type ABC transporter 2 (Fragment) | |
|  | 1614987_at | CF609746 | 0.38 | MATE efflux family protein 1 | |
|  | 1609370_at | CB972302 | 0.38 | Hypothetical protein | |
|  | 1621529_at | CB344685 | 0.38 | OSJNBa0089K21.6 protein | |
|  | 1621011_at | BQ800185 | 0.37 | Putative sucrose transporter | |
|  | 1615697_at | AF021810.1 | 0.37 | Putative sucrose transporter | |
|  | 1617554_at | CF414932 | 0.37 | Putative beta-subunit of K+ channels | |
|  | 1610744_at | CF415597 | 0.36 | Ammonium transporter (AMT1.1) | |
|  | 1610839_at | CB982193 | 0.34 | Peroxisomal adenine nucleotide carrier 1 | |
|  | 1619541_at | CB979469 | 0.34 | Plasma membrane H+-ATPase (Fragment) | |
|  | 1616083_at | CB009608 | 0.33 | Hexose transporter | |
|  | 1610437_at | CF210626 | 0.33 | Nitrate transporter NTL1 | |
|  | 1616720_at | BQ800605 | 0.32 | Putative short-chain acyl-CoA oxidase | |
|  | 1616662_at | CF404703 | 0.32 | Potential calcium-transporting ATPase 13 | |
|  | 1622128_s_at | CD798482 | 0.32 | Putative aquaporin TIP3 | |
|  | 1616707_at | CF604179 | 0.31 | Tetracycline transporter protein-like | |
|  | 1622021_at | CF209010 | 0.31 | NHX1 | |
|  | 1606834_s_at | CB968939 | 0.30 | Putative mitochondrial carrier protein | |
|  | 1615295_at | CF373832 | 0.30 | Putative lipid transfer protein GPI-anchored | |
|  | 1611326_at | CF513404 | 0.29 | Putative sugar transporter | |
|  | 1619989_s_at | BQ792762 | 0.28 | ADP,ATP carrier protein | |
|  | 1617575_s_at | CD800130 | 0.28 | Thioredoxin H | |
|  | 1607817_at | BQ792658 | 0.28 | Putative purine permease | |
|  | 1612331_at | CB344631 | 0.28 | Putative amino acid transport protein AAP2 | |
|  | 1617609_at | CF207468 | 0.28 | EspB-like protein | |
|  | 1618607_s_at | CB972339 | 0.26 | Zinc transporter | |
|  | 1608259_at | CF205412.1 | 0.26 | Zinc transporter | |
|  | 1617662_at | BQ794604 | 0.25 | Thioredoxin 2 | |
|  | 1618605_at | CB342936 | 0.24 | Mitochondrial carrier protein, putative | |
|  | 1616788_at | CF202817.1 | 0.24 | Nitrate transporter | |
|  | 1610123_at | CB009127 | 0.23 | Potassium transporter 4 ( | |
|  | 1619984_at | CF519097 | 0.22 | Amino acid carrier | |
|  | 1622191_at | CD801608 | 0.21 | Sulfate transporter 2 | |
|  | 1613896_at | CA818837 | 0.19 | Nitrate transporter NRT1-2 | |
|  | 1609917_at | CB917882 | 0.17 | Putative mitochondrial dicarboxylate carrier protein | |
|  | 1607432_s_at | AF188843.1 | 0.17 | Plasma membrane aquaporin | |
|  | 1609973_at | CD801577 | 0.17 | Putative ABC transporter | |
|  | 1619168_at | CF405126 | 0.17 | Putative mitochondrial dicarboxylate carrier protein | |
|  | 1618589_s_at | CF206361.1 | 0.15 | Actinorizal nodulin AgNOD-GHRP | |
|  | 1622416_at | CF518913 | 0.09 | Lipid transfer protein | |
| Energy | 1621495_at | CF515274 | 4.70 | Carbonic anhydrase | |
|  | 1607627_at | CD720153 | 2.66 | NADH-plastoquinone oxidoreductase subunit 2 | |
|  | 1614266_at | BQ792322 | 2.64 | Cytochrome c heme attachment protein | |
|  | 1617605_at | CF513977 | 2.52 | Photosystem II CP47 protein (Fragment) | |
|  | 1608000_at | CF517275 | 2.26 | Photosystem II protein D2 | |
|  | 1619754_at | BQ800202 | 0.47 | Putative cytochrome c oxidase subunit Vb ( | |
|  | 1617444_s_at | CF373801 | 0.47 | Lipoxygenase (Fragment) | |
|  | 1608892_at | CF373801 | 0.45 | Lipoxygenase (Fragment) | |
|  | 1619850_at | CD717468 | 0.45 | Aconitate hydratase | |
|  | 1607800_at | CB972521 | 0.44 | Putative cytochrome c oxidoreductase | |
|  | 1608100_at | CF404013 | 0.44 | PEP carboxylase | |
|  | 1622514_at | CA815335 | 0.41 | Phosphoglycerate mutase family, putative | |
|  | 1612018_at | CF208964 | 0.39 | Glucose-6-phosphate isomerase | |
|  | 1622074_at | BQ794083 | 0.39 | Phosphoenolpyruvate carboxylase kinase 2 | |
|  | 1610699_at | CF518312 | 0.37 | Putative cytochrome c biogenesis protein | |
|  | 1611004_at | CF510607 | 0.33 | Early nodulin-like protein 15 | |
|  | 1609975_at | CB342865 | 0.26 | Putative mitochondrial energy transfer protein | |
|  | 1618277_at | CF568829 | 0.13 | Glyceraldehyde-3-phosphate dehydrogenase precursor | |
| Protein synthesis | 1620244_at | CD720153 | 3.78 | NADH-plastoquinone oxidoreductase subunit 2 | |
|  | 1619536_at | CB971140 | 2.48 | Putative translation releasing factor 2 | |
|  | 1617618_at | CD798691 | 2.32 | Ribosomal proteins | |
|  | 1618321_at | CB982883 | 2.24 | Putative Ligatin | |
|  | 1617056_at | CF214470 | 0.46 | Ribosomal protein S29 (Fragment) | |
| Cell cycle and DNA processing | 1617240_at | CF510707 | 4.19 | putative histone acetyltransferase | |
|  | 1607336_at | BQ800260 | 0.46 | Actin-depolymerizing factor (ADF) | |
|  | 1612752_at | CF201925.1 | 0.44 | HMG protein | |
|  | 1618511_at | CF404182 | 0.41 | Hypothetical protein P0605D08.41 | |
|  | 1621077_s_at | CB975305 | 0.26 | High mobility group B protein 1 | |
|  | 1614635_at | CF519142 | 0.25 | Cell Division Protein AAA ATPase family | |
| Interaction with environment | 1617541_s_at | CB342503 | 6.14 | Beta-carotene hydroxylase 2 (Fragment) | |
|  | 1608814_at | CK138208.1 | 3.77 | Beta-carotene hydroxylase | |
|  | 1613054_at | BQ794856 | 0.45 | Putative auxin-regulated protein | |
|  | 1620281_at | CF608629 | 0.39 | Putative aldehyde dehydrogenase | |
|  | 1617572_at | CB918599 | 0.38 | BRH1 RING finger protein | |
|  | 1606788_s_at | CF204720.1 | 0.38 | Nine-cis-epoxycarotenoid dioxygenase 1 | |
|  | 1608502_at | BQ794120 | 0.37 | 12-oxophytodienoate reductase | |
|  | 1606557_at | CB976051 | 0.37 | Somatic embryogenesis receptor kinase 1 | |
|  | 1621326_at | CF606133 | 0.34 | Gibberellin 2-oxidase 1 | |
|  | 1620662_at | CB981820 | 0.32 | Auxin and ethylene responsive GH3-like protein | |
|  | 1607972_s_at | CF608629 | 0.32 | Putative aldehyde dehydrogenase | |
|  | 1619862_at | CB979196 | 0.30 | methyltransferase PMT21 | |
|  | 1607778_at | CA814507 | 0.30 | Gibberellin 2-oxidase 1 | |
|  | 1614660_at | CF207466 | 0.30 | Auxin-induced protein AUX22 | |
|  | 1608022_at | BQ798105 | 0.30 | 9-cis-epoxycarotenoid dioxygenase 1 | |
|  | 1618465_at | CB918507 | 0.29 | Shaggy-related protein kinase gamma | |
|  | 1607222_at | CF568970 | 0.28 | Putative aldehyde dehydrogenase | |
|  | 1620809_at | CF404050 | 0.28 | Hypothetical protein F26K9_160 | |
|  | 1618875_s_at | CF214574 | 0.28 | Auxin-responsive protein IAA26 | |
|  | 1611979_at | CB979128 | 0.27 | ERD3 protein | |
|  | 1606794_at | CB972023 | 0.25 | Thaumatin-like protein | |
|  | 1619610_at | CB008850 | 0.22 | IAA-amino acid hydrolase | |
|  | 1609995_s_at | CB970406 | 0.20 | 1-aminocyclopropane-1-carboxylate oxidase | |
|  | 1610607_at | CF371650 | 0.19 | 1-aminocyclopropane-1-carboxylate oxidase | |
|  | 1616698_at | CF201799.1 | 0.17 | 1-aminocyclopropane-1-carboxylate oxidase | |
|  | 1622147_at | CF604303 | 0.16 | 1-aminocyclopropane-1-carboxylate oxidase 3 | |
|  | 1621989_at | CD006576 | 0.14 | ABA 8'-hydroxylase CYP707A1 | |
|  | 1620276_at | CF605672 | 0.12 | Putative embryo-abundant protein | |
|  | 1616695_s_at | BQ795049 | 0.10 | Thaumatin | |
| Interaction with cellular environment | 1610316_at | CD799041 | 0.36 | Putative COPT5 | |
| Transposable elements | 1620073_at | CB002740 | 23.27 | Polyprotein | |
| Protein activity regulation | 1607683_at | CF207162 | 0.42 | Serpin-like protein (Fragment) | |
| Prtein with binding function | 1610078_at | CF208751 | 2.17 | Zinc finger protein-like | |
|  | 1616601_at | CF210956 | 0.45 | Putative RNA binding protein | |
|  | 1607745_at | CB009252 | 0.45 | Sequence-specific DNA binding transcription factor | |
|  | 1615828_at | CF209855 | 0.43 | Ttg1-like protein | |
|  | 1607049_s_at | CF514518 | 0.43 | Type 2 metallothionein | |
|  | 1616319_at | AY150521.1 | 0.42 | Putative heterogeneous nuclear ribonucleoprotein | |
|  | 1616684_at | CD797892 | 0.41 | Hypothetical protein | |
|  | 1617167_at | CB343765 | 0.41 | KH domain-containing protein (Fragment) | |
|  | 1610259_at | CF209770 | 0.41 | Ttg1-like protein | |
|  | 1607163_at | CF415171 | 0.35 | Type 2 metallothionein | |
|  | 1613521_at | CF514518 | 0.35 | Type 2 metallothionein | |
|  | 1615950_s_at | CF371996 | 0.33 | Poly(A)-binding protein (Fragment) | |
|  | 1613096_at | CA816045 | 0.33 | Zinc finger CCCH domain-containing protein 24 | |
|  | 1621900_s_at | CD798424 | 0.31 | Similarity to RNA binding protein | |
|  | 1616920_at | CF512566 | 0.26 | Nucleic acid binding protein-like | |
|  | 1614725_at | CA817463 | 0.25 | DNA-binding protein 4 | |
|  | 1613528_at | CF513262 | 0.16 | Protein SKU5 similar 17 | |
| Cell fate | 1609909_s_at | CF206328.1 | 4.91 | Putative xyloglucan endotransglycosylase/hydrolase (Fragment) | |
|  | 1616312_at | CD720049 | 4.60 | Putative phytosulfokine peptide precursor | |
|  | 1620608_s_at | CD801046 | 0.46 | RALF | |
|  | 1617739_at | CB976948 | 0.40 | Xyloglucan endotransglycosylase precursor | |
|  | 1619082_at | CF215502 | 0.37 | Expansin 3 | |
|  | 1620840_at | CB968965 | 0.37 | Putative alpha-expansin | |
|  | 1622456_at | CF609276 | 0.27 | Putative phytosulfokine peptide precursor | |
|  | 1619010_s_at | BQ794765 | 0.21 | Expansin | |
|  | 1620443_s_at | CF609276 | 0.20 | Putative phytosulfokine peptide precursor | |
|  | 1617372_x_at | CF201816.1 | 0.15 | Putative phytosulfokine peptide precursor | |
|  | 1608074_s_at | CF215793 | 0.14 | Expansin | |
|  | 1607170_s_at | CB917184 | 0.13 | Putative phytosulfokine peptide precursor | |
|  | 1612224_s_at | CF201816.1 | 0.10 | phytosulfokine peptide precursor | |
|  | 1613527_at | CB978490 | 0.10 | Alpha-expansin 3 | |
|  | 1612253_at | CB970527 | 0.09 | Expansin-2 | |
|  | 1610418_at | BQ798078 | 0.05 | Expansin | |
|  | 1621251_s_at | BQ795002 | 0.03 | Xyloglucan endo-transglycosylase | |
| Development | 1612840_a_at | BQ794471 | 0.44 | LOB domain protein 1 | |
|  | 1618306_at | CF605844 | 0.36 | Stigma-specific Stig1 family protein | |
|  | 1614607_at | BQ799173 | 0.27 | Putative senescence-associated protein (Fragment) | |
| Biogenesis of cellular components | 1618889_at | CB981513 | 2.57 | Pherophorin-dz1 protein precursor | |
|  | 1614832_at | CA808973 | 2.21 | Putative heat-shock protein related cluster | |
|  | 1620864_at | CF403974 | 0.42 | NT3 | |
|  | 1616561_at | CB982529 | 0.41 | Reversibly glycosylated polypeptide | |
|  | 1615469_at | CB974963 | 0.37 | Extensin (Class I) | |
|  | 1622234_s_at | CA814443 | 0.35 | Reversibly glycosylated polypeptide | |
|  | 1615708_s_at | CF605377 | 0.35 | Putative membrane protein precursor | |
|  | 1606756_at | CB005678 | 0.30 | Alpha-1,4-glucan-protein synthase [UDP-forming] 1 | |
|  | 1606478_at | CB346797 | 0.30 | Putative myosin heavy chain | |
|  | 1610563_at | CB339863 | 0.29 | Putative myosin heavy chain | |
|  | 1608706_at | CF404110 | 0.28 | Putative serine/proline-rich protein | |
|  | 1622084_at | CF513595 | 0.27 | Kinesin-like protein; 73641-79546 | |
|  | 1607449_s_at | AY046416.1 | 0.24 | Proline-rich protein 1 | |
|  | 1614803_at | AY046416.1 | 0.22 | Proline-rich protein 1 | |
|  | 1621384_at | AY046417.1 | 0.20 | Proline rich protein 2 | |
|  | 1619401_at | CF373107 | 0.15 | Arabinogalactan peptide 20 | |
|  | 1607162_s_at | CF512517 | 0.11 | Proline-rich-like protein | |
|  | 1610311_at | CF373485 | 0.11 | TED3 | |
|  | 1617023_at | CF210510 | 0.10 | Hybrid proline-rich protein | |
|  | 1619613_at | CD801720 | 0.09 | Hybrid proline-rich protein | |
| Storage protein | 1613038_s_at | CA818530 | 2.35 | Seed maturation protein PM37 | |
|  | 1610600_at | CA813949 | 0.38 | Seed specific protein Bn15D18B | |
