# Supplementary material for: Transcriptomic analysis of grape (Vitis vinifera L.) leaves during and after recovery from heat stress
Source: BMC Plant Biol. 2012 Sep 28;12:174. doi: 10.1186/1471-2229-12-174 (PMC3497578; doi:10.1186/1471-2229-12-174)
Supplement: Additional file 8 — Genes upregulated or downregulated unique to the recovery in grapevine leaves. [file 1471-2229-12-174-S8.docx]

**Additional file 8 Genes upregulated or downregulated unique to the recovery in grape leaves**

| **Category** | **Probe sets** | **Accession** | **Fold change** | **Gene name description** |
| --- | --- | --- | --- | --- |
| Cell rescue | 1613471_at | CF215857 | 7.17 | Putative pathogenesis-related protein |
|  | 1612444_at | CF214623 | 4.25 | MLP-like protein 28 |
|  | 1617579_at | CD713565 | 3.01 | Late embryogenesis abundant protein D-29 |
|  | 1619164_at | CB344468 | 2.98 | RD22-like protein |
|  | 1610521_at | CF403314 | 2.92 | Major latex-like protein |
|  | 1612707_at | CD801530 | 2.64 | Fe-SOD |
|  | 1621587_at | CF214129 | 2.63 | Adenylyl-sulfate reductase precursor |
|  | 1614543_a_at | CF517472 | 0.45 | Enhanced protein 2 |
|  | 1620043_at | AY427149.1 | 0.43 | Resistance protein candidate |
|  | 1622142_at | CB839449 | 0.41 | Avr9/Cf-9 rapidly elicited protein 256 |
|  | 1622652_at | CD004838 | 0.40 | Similarity to SRC2 protein |
|  | 1616706_at | CF207563 | 0.40 | Stress-induced protein sti1-like protein |
|  | 1612826_at | AJ536326.1 | 0.38 | Putative pathogenesis related protein 1 precursor |
|  | 1609971_at | CD004838 | 0.35 | Similarity to SRC2 protein |
|  | 1613154_at | CB346750 | 0.33 | Putative stress-induced protein sti1 |
|  | 1622550_at | AY427148.1 | 0.32 | Resistance protein candidate (Fragment) |
|  | 1618110_s_at | CF404940 | 0.32 | Aluminum induced protein with YGL  and LRDR motifs |
|  | 1618778_at | CA814441 | 0.30 | Avr9/Cf-9 rapidly elicited protein 75 |
|  | 1609037_at | CB342383 | 0.28 | Avr9/Cf-9 rapidly elicited protein 140 |
|  | 1614141_at | CA817693 | 0.20 | Expressed protein |
|  | 1611876_s_at | CF202444.1 | 0.19 | Chitinase 3-like protein |
|  | 1621881_at | CF404940 | 0.18 | Aluminum induced protein with YGL  and LRDR motifs |
|  | 1608868_at | AY427142.1 | 0.18 | Resistance protein candidate |
|  | 1607408_at | CF216013 | 0.14 | Protein Nimi-interacting1 |
|  | 1611058_at | CA814153 | 0.10 | Putative pathogenesis related protein 1 precursor |
|  | 1622015_at | CF605567 | 0.09 | Glutathione S-transferase 12 |
|  | 1606770_s_at | CF202828.1 | 0.07 | Rhizome dirigent protein |
|  | 1618663_s_at | BM436446 | 0.07 | Disease resistance response protein-like |
|  | 1618326_x_at | CF205664.1 | 0.03 | Disease resistance response protein-like |
|  | 1612858_at | CF205163.1 | 0.02 | Putative pathogenesis related protein 1 precursor |
| Prtotein fate | 1612531_at | CF404764 | 11.25 | Eukaryotic aspartyl protease family protein |
|  | 1606741_at | CF200678.1 | 2.77 | U-box protein |
|  | 1608052_s_at | CB981502 | 0.48 | Hsp90-2 |
|  | 1618009_at | CD714666 | 0.45 | Hsp80 |
|  | 1619202_s_at | CB347488 | 0.45 | Putative luminal binding protein |
|  | 1615587_at | CF207514 | 0.44 | DnaJ-like protein |
|  | 1614594_at | CF518547 | 0.42 | F19K16.12 (Putative heat-shock protein) |
|  | 1619931_s_at | CB349799 | 0.42 | Hsp90-1 |
|  | 1608448_at | BQ792197 | 0.40 | Endoplasmin homolog precursor |
|  | 1615800_at | CA814508 | 0.39 | Heat shock transcription factor (HSF family) |
|  | 1615660_at | CF215672 | 0.39 | Ubiquitin-conjugating enzyme E2-17 kDa |
|  | 1616246_at | CF207965 | 0.35 | Hsc70-3 |
|  | 1614205_at | CF404132 | 0.34 | Putative zinc finger protein |
|  | 1612310_at | CA815999 | 0.34 | NtHSF1 |
|  | 1609222_at | CF518991 | 0.30 | Hsp22 |
|  | 1619542_at | CF204970.1 | 0.29 | OSJNBa0022H21.7 protein |
|  | 1610896_at | CF204993.1 | 0.29 | Grp94 |
|  | 1619528_s_at | CF215279 | 0.28 | Luminal binding protein 5 precursor |
|  | 1607002_at | CB347488 | 0.27 | Putative luminal binding protein |
|  | 1608762_at | BQ797424 | 0.27 | HSF7 |
|  | 1616357_at | CF516994 | 0.26 | Protein kinase domain-containing protein |
|  | 1609949_at | CF373330 | 0.23 | Hsp70-2 |
|  | 1613042_at | CB346341 | 0.12 | NtHSP18 |
|  | 1612094_at | CF513922 | 0.08 | Hsp101 |
|  | 1616317_at | CD720960 | 0.08 | Miraculin homologue |
|  | 1616538_at | CB347766 | 0.05 | HSP17.4 |
|  | 1608429_at | BQ793090 | 0.04 | BCL-2-associated athanogene 5 |
|  | 1608164_at | CD008061 | 0.01 | HSP17.9 |
| Metabolism | 1619065_at | CB972322 | 5.01 | Putative cinnamoyl CoA reductase |
|  | 1615198_at | CF209943 | 4.34 | Alpha-glucosidase |
|  | 1612386_at | CB974139 | 3.97 | Cytochrome P-450-like protein |
|  | 1614485_at | CF207460 | 3.71 | Putative anthranilate N-hydroxycinnamoyl/ benzoyltransferase |
|  | 1617970_at | CF372887 | 3.66 | Putative anthocyanidine rhamnosyl-transferase |
|  | 1622891_at | CF372753 | 3.59 | Putative beta-1,3-glucanase |
|  | 1618112_at | CB971725 | 3.38 | Putative anthocyanidin-3-glucoside rhamnosyltransferase |
|  | 1612672_at | CF215975 | 3.36 | Pectin methyl esterase |
|  | 1621517_s_at | CF204966.1 | 3.30 | DC1.2 homologue |
|  | 1609765_s_at | CB007712 | 3.05 | Leucoanthocyanidin dioxgenase |
|  | 1613113_at | CK136925.1 | 2.93 | Phenylalanine ammonia lyase |
|  | 1617320_at | CB343024 | 2.90 | DC1.2 homologue |
|  | 1607147_at | CF404016 | 2.68 | Cytosolic alpha-amylase |
|  | 1607374_at | CF404162 | 2.67 | Beta-D-glucosidase |
|  | 1621307_at | CB969961 | 2.60 | Prephenate dehydratase |
|  | 1607272_at | CF215703 | 2.59 | Glucosyltransferase-like protein |
|  | 1607848_at | CB339435 | 2.59 | Inorganic pyrophosphatase 1 |
|  | 1620679_at | CB972076 | 2.54 | Beta-glucosidase, putative |
|  | 1618510_at | CF203363.1 | 2.53 | Probable hydroquinone glucosyltransferase |
|  | 1609932_at | CB970042 | 2.50 | Prephenate dehydratase |
|  | 1611081_a_at | BQ799201 | 2.40 | Putative polygalacturonase |
|  | 1617875_at | CB971740 | 2.33 | F5I14.13 protein |
|  | 1615481_at | CB973026 | 2.29 | Cytochrome b5 DIF-F |
|  | 1611739_at | CF403783 | 2.19 | Putative cytochrome P450 |
|  | 1613165_s_at | CB972844 | 2.15 | L-idonate dehydrogenase |
|  | 1615574_at | CB977067 | 2.13 | Pectin methylesterase isoform alpha |
|  | 1613760_at | CF204137.1 | 0.45 | Alpha-amylase |
|  | 1620994_at | CD802275 | 0.45 | LRK33 |
|  | 1617293_s_at | BQ792635 | 0.43 | CIG1 |
|  | 1622701_at | CF415421 | 0.43 | Progesterone 5-beta-reductase |
|  | 1621826_at | CB975558 | 0.42 | BON1-associated protein 2 |
|  | 1610410_at | CB342966 | 0.41 | Putative glucosyltransferase |
|  | 1622752_at | CF214629 | 0.41 | Putative serine hydrolase |
|  | 1609270_at | CF209679 | 0.40 | ACT domain repeat 8 protein |
|  | 1606798_at | CF209842 | 0.40 | Glutamine synthetase cytosolic isozyme 2 |
|  | 1616014_at | CB973891 | 0.40 | Putative pyridine nucleotide-disulphide oxidoreductase |
|  | 1611135_at | CB983077 | 0.39 | Putative alpha-hydroxynitrile lyase |
|  | 1620283_s_at | CB916572 | 0.39 | Alpha-amylase |
|  | 1608225_s_at | CF209285 | 0.38 | Putative alpha-hydroxynitrile lyase |
|  | 1611897_s_at | CB347033 | 0.38 | Caffeoyl-CoA-O-methyltransferas |
|  | 1618373_at | Z68123.1 | 0.37 | Acidic endochitinase precursor |
|  | 1613619_at | CF209453 | 0.36 | Cytochrome like protein |
|  | 1622651_at | CF605906 | 0.35 | Polyphenol oxidase |
|  | 1618595_at | BQ798258 | 0.29 | (-)-isopiperitenol dehydrogenase |
|  | 1620347_at | CA814065 | 0.24 | Glycosyltransferase |
|  | 1609234_at | CF215945 | 0.24 | Polyphenol oxidase |
|  | 1611611_at | CB980376 | 0.21 | Germin-like protein precursor |
|  | 1619034_at | CK136955.1 | 0.18 | Putative cytochrome P450 |
|  | 1621363_at | CA809467 | 0.15 | Cytochrome P450 |
|  | 1610824_s_at | AY059639.1 | 0.12 | Stilbene synthase 3 |
|  | 1612804_at | X76892.1 | 0.12 | Stilbene synthase |
|  | 1622638_x_at | X76892.1 | 0.11 | Stilbene synthase |
|  | 1612706_at | CB002359 | 0.10 | Laccase-like protein |
|  | 1610850_at | S63225.1 | 0.07 | Stilbene synthase 1 |
|  | 1611190_s_at | AF274281.1 | 0.07 | Resveratrol synthase |
|  | 1609696_x_at | S63225.1 | 0.06 | Stilbene synthase 1 |
|  | 1607438_at | CF205386.1 | 0.06 | Putative ripening-related protein |
|  | 1620964_s_at | S63225.1 | 0.06 | Stilbene synthase 1 |
|  | 1608026_at | CB982380 | 0.01 | Germin-like protein subfamily 1  member 15 precursor |
| Transcription | 1618519_at | CF214731 | 2.37 | Putative zinc finger protein |
|  | 1618504_at | CF512926 | 2.23 | Myc-like anthocyanin regulatory protein |
|  | 1609148_at | CF213127 | 2.20 | Squamosa promoter binding-like protein |
|  | 1615012_s_at | CB341464 | 0.46 | OSJNBa0086O06.18 protein |
|  | 1609555_at | CD719835 | 0.45 | NAC domain protein |
|  | 1609171_at | CA813062 | 0.40 | F22G5.9 |
|  | 1614332_s_at | CA814568 | 0.40 | ZPT2-14; similar to At ZAT12 |
|  | 1622399_at | CF206628.1 | 0.39 | DNA-binding protein WRKY3 |
|  | 1611285_s_at | CA809190 | 0.38 | WRKY transcription factor 68 |
|  | 1620817_at | CB341464 | 0.38 | OSJNBa0086O06.18 protein |
|  | 1611550_at | CD800189 | 0.36 | Putative WRKY transcription factor 30 |
|  | 1612448_at | CA817520 | 0.24 | NAC domain protein NAC6 |
|  | 1613141_at | CF518362 | 0.20 | Putative NAM (No apical meristem) protein |
|  | 1612781_at | CB978740 | 0.15 | Squalene monooxygenase |
| Signal transduction | 1617247_at | CB973852 | 4.13 | Protein ralf-like 34 |
|  | 1619795_at | CF403136 | 2.36 | Similarity to receptor protein kinase |
|  | 1611166_at | CF206128.1 | 0.47 | Putative leucine-rich repeat protein |
|  | 1614799_at | CF212957 | 0.43 | Putative calmodulin-binding protein |
|  | 1612466_at | CF373473 | 0.40 | Calcium binding protein |
|  | 1618364_at | CF514983 | 0.38 | GTPase activating protein-like |
|  | 1620562_at | CB343617 | 0.38 | Putative calmodulin-binding protein |
|  | 1617279_at | BQ796438 | 0.35 | Putative calmodulin-like protein |
|  | 1611917_at | CB972164 | 0.31 | Calnexin homolog precursor |
|  | 1620324_at | CB002490 | 0.28 | Probable LRR receptor-like serine |
|  | 1611127_at | CF510878 | 0.26 | Calmodulin-like protein |
|  | 1607701_at | CB002490 | 0.23 | Probable LRR receptor-like serine |
|  | 1609226_at | CF603976 | 0.21 | Calnexin homolog precurso |
|  | 1611918_s_at | CF603976 | 0.21 | Calnexin homolog precurso |
|  | 1619500_at | CF404052 | 0.20 | Receptor protein kinase-like protein |
| Transport regulation | 1607943_at | CB971933 | 15.95 | Putative aquaporin TIP3 |
|  | 1615829_s_at | CF371656 | 10.56 | Aquaporin |
|  | 1615415_s_at | BQ797891 | 3.54 | Plasma intrinsic protein 2,2 |
|  | 1612313_at | CF205543.1 | 3.41 | Amino acid permease 6 |
|  | 1615359_at | CA817149 | 3.34 | Nitrate transporter NRT1-5 |
|  | 1612325_at | CF403395 | 3.25 | Cytochrome P450-like protein |
|  | 1617400_at | BQ798101 | 3.19 | Sulfate transporter 3.1 |
|  | 1614158_at | CF513796 | 3.08 | Putative nitrite transporter |
|  | 1610982_at | CF214253 | 3.02 | MipC |
|  | 1608175_at | CF404148 | 2.76 | Lipid transfer protein |
|  | 1614034_at | CF205708.1 | 2.68 | Nitrate transporter |
|  | 1606656_at | CF212763 | 2.66 | P-rich protein NtEIG-C29 |
|  | 1608623_s_at | CF205543.1 | 2.62 | Amino acid permease 6 |
|  | 1611159_at | CF512047 | 2.60 | Sulphate transporter |
|  | 1618261_at | CF403474 | 2.56 | Probable nitrite transporter |
|  | 1618590_at | CF372692 | 2.41 | Putative ABC transporter protein |
|  | 1622822_at | CF519123 | 2.28 | Tonoplast intrinsic protein |
|  | 1615319_s_at | CF207944 | 2.17 | Nitrate transporter |
|  | 1622620_at | BQ798306 | 0.44 | Putative ABC transporter protein |
|  | 1613916_at | CF404702 | 0.44 | Isp4 protein-like |
|  | 1619140_at | CF606031 | 0.41 | Similar to Leishmania major |
|  | 1622786_at | CF207035.1 | 0.41 | SNAP25 homologous protein SNAP33 |
|  | 1611957_s_at | CD799692 | 0.41 | Amino acid transport protein AAT1 |
|  | 1615318_at | CF405245 | 0.36 | Potassium transporter 5 |
|  | 1607926_at | CF202256.1 | 0.34 | Putative membrane protein |
|  | 1610949_s_at | CF206451.1 | 0.34 | Heavy metal transport |
|  | 1608566_s_at | CA817781 | 0.34 | heavy metal ion transport |
|  | 1618132_at | CB976959 | 0.33 | Nitrate transporter NTL1 |
|  | 1620245_at | CF202722.1 | 0.30 | Cytochrome P450 |
|  | 1620992_at | CF608881 | 0.27 | Heavy metal transport/detoxification domain-containing protein |
|  | 1610914_at | CF203251.1 | 0.21 | Copper transporter 1 |
| Engery | 1620654_at | CF514699 | 2.87 | Transketolase 1 |
|  | 1617741_at | BQ797028 | 2.24 | Malate dehydrogenase precursor |
|  | 1615814_at | CB920915 | 0.17 | Glyceraldehyde-3-phosphate dehydrogenase |
| Prtotein synthesis | 1615542_at | CF415517 | 4.80 | Translation initiation factor IF2 |
| Cell cycle and DNA processing | 1610096_at | CF212486 | 2.55 | Histone H4 |
|  | 1608380_at | CF372703 | 2.53 | Hist1h4h protein |
|  | 1608305_at | CF415367 | 2.23 | Putative seed specific protein |
| Interaction with environment | 1616961_at | CF415013 | 2.48 | Gibberellin oxidase-like protein |
|  | 1615971_a_at | CB980630 | 2.14 | Heavy metal transporter |
|  | 1609591_at | CD799271 | 0.45 | Auxin responsive SAUR protein |
|  | 1613811_a_at | CB920849 | 0.35 | Putative fatty acid elongase |
|  | 1610623_s_at | CD802108 | 0.34 | Putative membrane protein |
|  | 1621371_at | CF202171.1 | 0.30 | Similarity to disease resistance response protein |
|  | 1620574_s_at | CF405646 | 0.25 | Probable auxin efflux carrier component 6 |
| Protein activity regulation | 1611666_s_at | AY156047.1 | 0.37 | Protease inhibitor |
| Protein with binding function | 1614505_s_at | CB975513 | 0.45 | F-box protein SKIP27 |
|  | 1612409_at | CB345904 | 0.26 | Calcium binding protein |
| Cell fate | 1622108_at | CF405863 | 2.54 | Putative monocopper oxidase precursor |
| Development | 1618774_at | CF415442 | 2.42 | Lateral organ boundaries-like 1 |
|  | 1608493_at | CF516947 | 2.27 | SAH7 protein |
|  | 1616413_at | AF003007.1 | 0.41 | Pathogenesis-related protein R |
|  | 1614047_s_at | CF512458 | 0.38 | Putative seed imbibition protein |
|  | 1611296_at | BM437675 | 0.36 | Senescence-inducible chloroplast stay-green protein 1 |
| Biogenesis of cellular components | 1607541_at | CF208308 | 3.78 | EP-repeat protein precursor |
|  | 1615201_at | CF415287 | 3.73 | Putative proline-rich protein |
|  | 1611976_at | CF213700 | 2.46 | Putative extensin |
|  | 1622152_at | CD802381 | 0.46 | Dynein light chain |
|  | 1606588_s_at | CA811171 | 0.32 | Tubulin alpha-6 chain |
|  | 1609447_at | CA811171 | 0.32 | Tubulin alpha-6 chain |
|  | 1615789_at | BQ795769 | 0.17 | Extensin precursor |
| Storage protein | 1607320_s_at | CF203023.1 | 0.07 | Globulin-like protein |
